# Dataset of asymmetric intramolecular [4+3] cycloaddition reactions catalyzed by NHC-gold(I) complexes

**DOI:** 10.1016/j.dib.2019.104409

**Published:** 2019-08-21

**Authors:** Ruoyu Ma, Jianbo Yang, Steven Kelley, Benjamin W. Gung

**Affiliations:** a Department of Chemistry & Biochemistry, Miami University, Oxford, OH 45056, USA; b Elmer O. Schlemper X-ray Diffraction Center, University of Missouri-Columbia, Columbia, Mo 65211, USA

**Keywords:** NHC-gold(I) complexes, Cycloaddition, Enantioselective, DFT calculation

## Abstract

The shared data is the unpublished portion of the experimental section for the article with the title “NHC–Au(I) catalyzed enantioselective intramolecular [4 + 3] cycloaddition of furan propargyl esters”.[1] The preparation of the intermediates for chiral NHC-gold(I) complexes and the furan propargyl ester substrates are included in this article. The ^1^H NMR and ^13^C NMR spectra of the gold complexes **17a-19c** and the X-ray crystal data of **17a**, **18a** and cycloaddition product **24** are also provided in this article or in Mendeley Data. Finally, the chiral HPLC spectra used to determine enantiomeric excess and Cartesian coordinates of the optimized structure of **25** and **26** calculated by DFT calculation are also presented in the article.

**Table:** Specifications Table

Subject	Organometallic chemistry, Asymmetric catalysis
Specific subject area	Asymmetric gold(I) catalysis, N-Heterocyclic carbene ligand, [4 + 3] cycloaddition, furan propargyl ester
Type of data	Tables of detailed X-ray crystal data and Cartesian coordinates Text files of experimental and NMR data Figures of compound structures, X-ray crystal packing pattern and chiral HPLC spectra
How data were acquired	NMR spectra were recorded on Bruker Av-500 and Av-300 instruments and calibrated by using residual undeuterated solvent as an internal reference (CHCl_3_: δ = 7.26 (^1^H), 77.16 ppm (^13^C)). Single crystal X-ray diffraction (SCXRD) for **18a** and **19a** were collected on a Bruker SMART diffractometer equipped with an Apex II area detector using Mo-Kα radiation from a fine-focus sealed source tube with a focusing collimator (Bruker Nano, Inc., Madison, WI). SCXRD data for **24** was collected on a Bruker D8 Venture diffractometer equipped with a Photon 100 CMOS area detector using Mo-Kα radiation from a microfocus source (Bruker Nano). Enantiomeric excess was determined by chiral HPLC.
Data format	Raw (Structures in ChemDraw) Analyzed (NMR data with assigned peaks, X-ray crystallography data, Cartesian coordinates of molecular structures, reaction conditions)
Parameters for data collection	All NMR spectra were collected with CDCl_3_ as solvent in 298K. For all crystals, hemispheres of data were collected using strategies of scans about the omega and phi axes with frame widths of 0.5°. Data collection, unit cell determination, data reduction, absorption correction, and scaling were performed using the Bruker Apex3 software suite: Apex3, AXScale and SAINT, version 2017.3-0; Bruker AXS Inc.: Madison, WI, 2017. Enantiomeric excess was determined by Lux® 5 μm Amylose-1 column (i-PrOH/hexane = 1/9, 0.5ml/min, 220nm)
Description of data collection	All NMR samples were dissolved in CDCl_3_ before running. All chiral HPLC samples were dissolved in a mixed solvent of i-PrOH/hexane = 1/9.
Data source location	Department of Chemistry and Biochemistry, Miami University, Oxford, Ohio, United States of America
Data accessibility	All data are either within the article or in public repository (NMR spectra) Repository name: Mendeley Data https://data.mendeley.com/datasets/zh4gp5x682/4
Related research article	Author's name: Ruoyu Ma, Jianbo Yang, Steven Kelley, Benjamin W. Gung Title: NHC–Au(I) catalyzed enantioselective intramolecular [4 + 3] cycloaddition of furan propargyl esters Journal: Journal of Organometallic Chemistry, Year: 2019, Volume: 898, DOI: https://doi.org/10.1016/j.jorganchem.2019.07.016

**Table:** 

**Value of the data** • The data in this article will be informative to synthesis community. • The data in this article will be beneficial to the researches who do asymmetric gold catalysis and [4 + 3] cycloaddition reactions. • The data in this article can be used in future design of the asymmetric gold catalysts and substrates. • The crystal structure of gold complex **17a** and **18a** are useful for the development of chiral NHC–Au(I) catalysts. The chiral HPLC data from the [4 + 3] cycloaddition reaction are useful for future development of these types of reactions.

## 1 Data

**Fig. 1:**
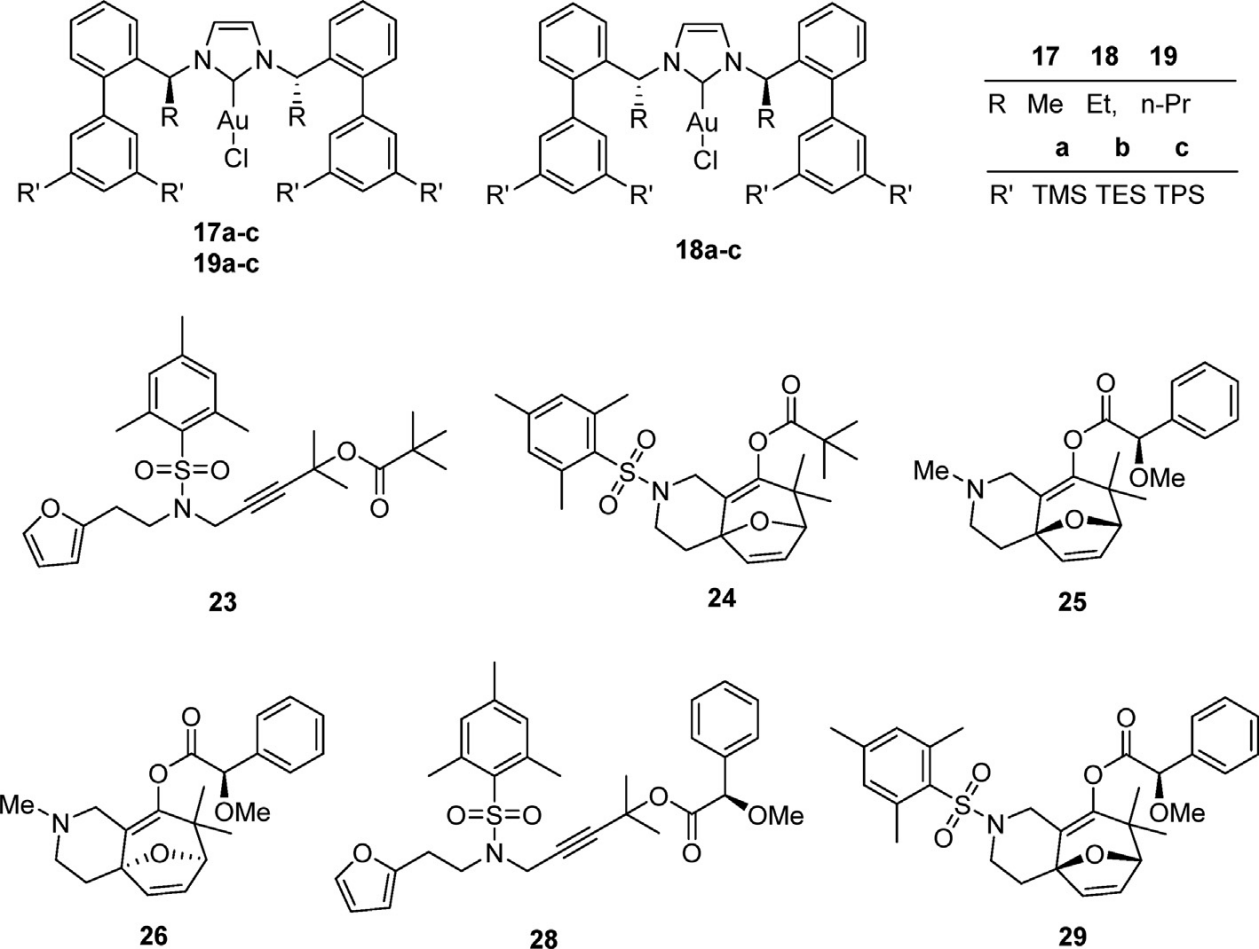
Structures of gold complexes **17a-19c** (TMS = Me_3_Si-; TES = Et_3_Si-; TPS = Pr_3_Si-), furan propargyl esters **23**, **28**, cycloaddition products **24**, **29** and DFT calculation models **25** and **26**.

The preparation and experimental data of the chiral sulfinamide intermediates for the gold complexes **17a-19c** and the furan propargyl esters **23** and **28** (Fig. 1) are presented in this article. NMR spectra of the gold complex **17a-19c** (Fig. 1) and cycloaddition product **24** and **29** (Fig. 1) are deposited in the repository of Mendeley Data (https://data.mendeley.com/datasets/zh4gp5x682/4). In each ^13^C NMR spectra of the gold complexes, a characteristic peak of the carbene carbon was observed with chemical shift at around 170 ppm.

**Fig. 2:**
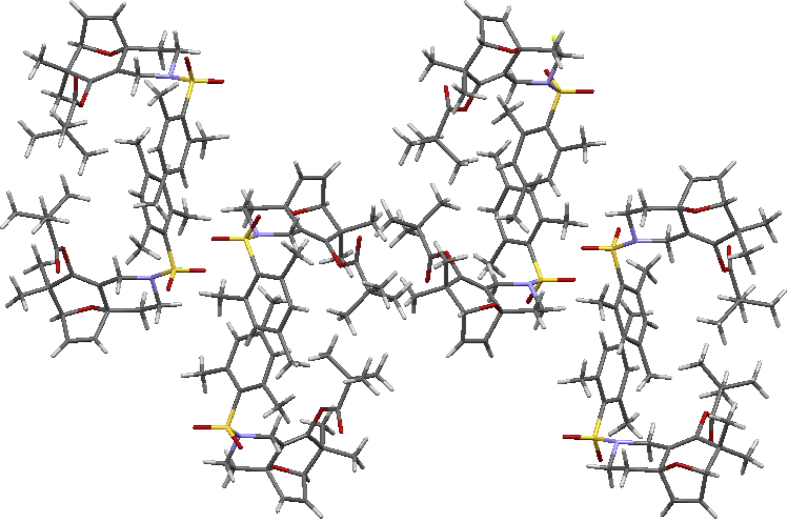
Crystal structure of the racemic cycloaddition product **24** with four pairs of enantiomers packing in a unit cell. The ester carbonyl group and the dihydrofuran double bond have a *syn*-relationship.

**Fig. 3:**
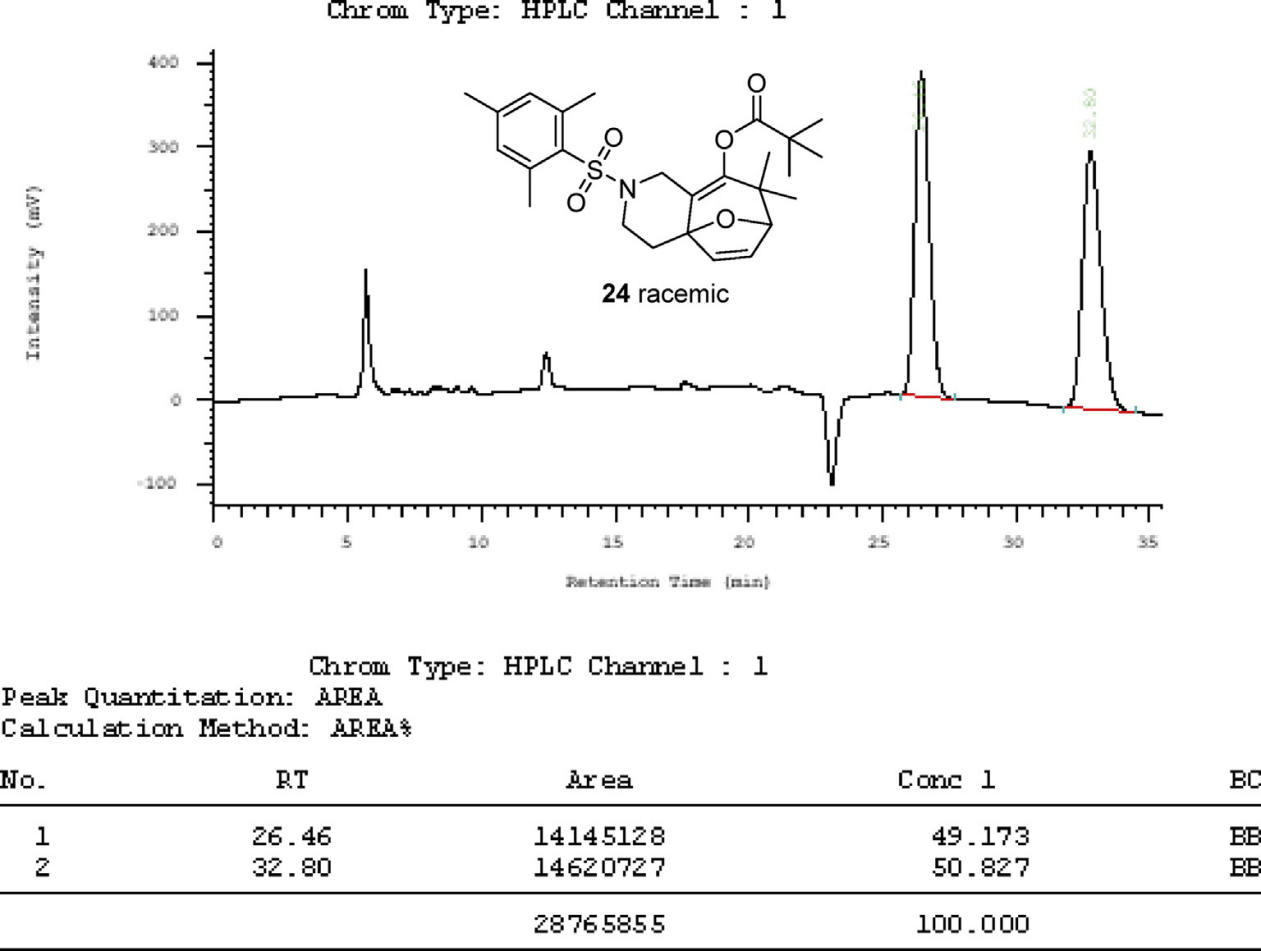
Chrial HPLC spectrum of racemic mixture of cycloaddition product **24.**

**Fig. 4:**
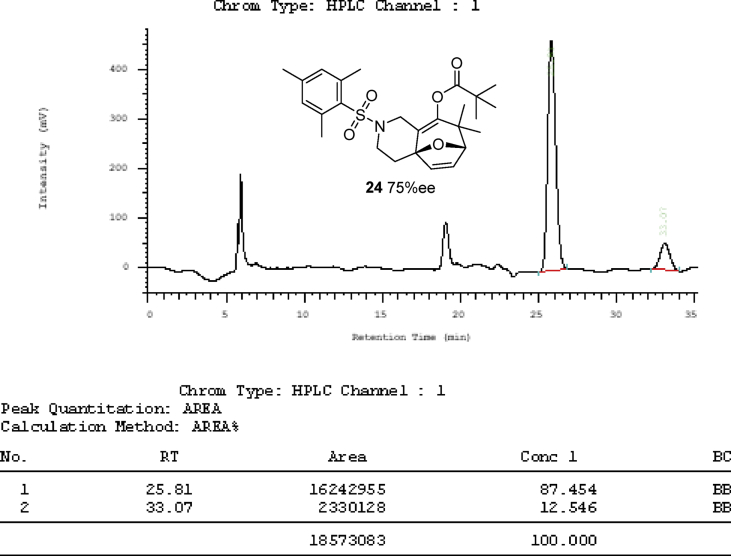
Chrial HPLC spectrum of cycloaddition product **24** with 75% ee.

The crystal structure of the racemic cycloaddition product **24** is shown in Fig. 2 (page 33) with four pairs of enantiomers packing in a unit cell. The ester carbonyl group and the dihydrofuran double bond have a *syn*-relationship. What also presented in article are the detailed X-ray crystal data including the bond length and bond angle of gold complex **17a**, **18a** and the cycloaddition product **24** (Table 1, Table 2, Table 3, Table 4, Table 5, Table 6, Table 7, Table 8, Table 9) and chiral HPLC spectra of cycloaddition products with racemic mixture (Fig. 3, page 39) and 75% enantiomeric excess (Fig. 4, page 40). Finally the Cartesian coordinates of the optimized structures of **25** and **26** calculated by density functional theory are presented in this article.

**Table 1:** Crystal data and structure refinement for **17a**.

Identification code	s1	
Empirical formula	C43H60 Au Cl N2 Si4	
Formula weight	949.70	
Temperature	173 K	
Wavelength	1.54178 Å	
Crystal system	Monoclinic	
Space group	P 1 21 1	
Unit cell dimensions	a = 13.5547 (11) Å	α = 90°.
	b = 9.6437 (8) Å	β = 101.828 (5)°.
	c = 18.5089 (16) Å	γ = 90°.
Volume	2368.1 (3) Å^3^	
Z	2	
Density (calculated)	1.332 Mg/m^3^	
Absorption coefficient	7.540 mm^−1^	
F (000)	968	
Crystal size	0.73 × 0.04 × 0.02 mm^3^	
Theta range for data collection	2.439–74.156°.	
Index ranges	−16 ≤ h<=16, −11 ≤ k<=11, −23 ≤ l<=23	
Reflections collected	85739	
Independent reflections	9485 [R (int) = 0.0579]	
Completeness to theta = 67.679°	100.0%	
Absorption correction	Semi-empirical from equivalents	
Max. and min. transmission	0.7538 and 0.4706	
Refinement method	Full-matrix least-squares on F^2^	
Data/restraints/parameters	9485/1/474	
Goodness-of-fit on F^2^	1.046	
Final R indices [I > 2sigma(I)]	R1 = 0.0282, w*R*2 = 0.0710	
R indices (all data)	R1 = 0.0303, w*R*2 = 0.0720	
Absolute structure parameter	0.039 (3)	
Extinction coefficient	n/a	
Largest diff. peak and hole	3.098 and −0.641 e.Å^−3^

**Table 2:** Atomic coordinates (x 10^4^) and equivalent isotropic displacement parameters (Å^2^x 10^3^) for **17a**. U (eq) is defined as one third of the trace of the orthogonalized U^ij^ tensor.

	x	y	z	U (eq)
Au (1)	944 (1)	2513 (1)	1269 (1)	28 (1)
Cl (1)	609 (1)	284 (2)	1566 (1)	40 (1)
Si(4)	2618 (1)	6068 (2)	3474 (1)	35 (1)
Si(3)	4632 (1)	1180 (2)	2805 (1)	36 (1)
C (1)	1152 (4)	4477 (6)	1005 (3)	28 (1)
C (32)	3869 (5)	4890 (7)	1673 (4)	31 (1)
C (37)	3384 (5)	5655 (7)	2142 (3)	33 (1)
C (36)	3252 (5)	5104 (7)	2819 (3)	34 (1)
C (34)	4129 (5)	2969 (6)	2554 (3)	33 (1)
C (33)	4218 (5)	3565 (7)	1884 (3)	34 (1)
C (35)	3621 (5)	3761 (7)	3004 (3)	34 (1)
C (38)	6015 (6)	1167 (9)	2814 (5)	54 (2)
C (40)	3984 (6)	−68 (8)	2083 (4)	42 (2)
C (42)	1459 (6)	5127 (8)	3566 (4)	45 (2)
C (39)	4401 (7)	721 (8)	3738 (4)	49 (2)
C (41)	3490 (6)	6093 (10)	4402 (4)	51 (2)
N (1)	1776 (4)	5011 (5)	602 (3)	27 (1)
C (2)	1619 (5)	6426 (6)	512 (3)	31 (1)
N (2)	617 (4)	5561 (5)	1185 (3)	26 (1)
C (3)	899 (5)	6779 (6)	877 (3)	30 (1)
C (26)	3435 (5)	5058 (6)	279 (3)	30 (1)
C (31)	3683 (5)	5521 (7)	−369 (4)	36 (1)
C (30)	4523 (5)	6357 (8)	−352 (4)	42 (2)
C (27)	4039 (4)	5458 (6)	956 (3)	31 (1)
C (24)	2494 (4)	4190 (6)	272 (3)	29 (1)
C (28)	4862 (5)	6328 (7)	961 (4)	38 (1)
C (29)	5099 (5)	6784 (8)	306 (4)	44 (2)
C (25)	1948 (5)	3677 (7)	−483 (4)	38 (1)
Si(2)	−3114 (1)	5782 (2)	3849 (1)	33 (1)
Si(1)	−596 (2)	1099 (2)	3596 (1)	38 (1)
C (14)	−1297 (4)	2711 (8)	3192 (3)	33 (1)
C (6)	−1197 (4)	4985 (6)	1036 (3)	26 (1)
C (10)	−2921 (5)	4190 (7)	827 (3)	32 (1)
C (17)	−2469 (4)	4942 (7)	2535 (3)	30 (1)
C (15)	−1828 (5)	3551 (7)	3595 (3)	32 (1)
C (13)	−1354 (5)	3031 (7)	2449 (3)	32 (1)
C (16)	−2418 (5)	4685 (7)	3280 (3)	32 (1)
C (9)	−3031 (5)	4412 (7)	75 (3)	33 (1)
C (5)	−360 (5)	6716 (6)	1998 (3)	31 (1)
C (11)	−2012 (4)	4457 (6)	1315 (3)	29 (1)
C (7)	−1308 (5)	5167 (6)	283 (3)	29 (1)
C (4)	−226 (4)	5406 (6)	1571 (3)	28 (1)
C (12)	−1929 (4)	4144 (6)	2112 (3)	29 (1)
C (8)	−2215 (5)	4888 (7)	−200 (3)	33 (1)
C (19)	772 (6)	1265 (8)	3593 (4)	46 (2)
C (23)	−4082 (5)	6833 (8)	3232 (4)	44 (2)
C (20)	−775 (8)	861 (9)	4564 (4)	58 (2)
C (21)	−2204 (7)	6966 (9)	4445 (4)	57 (2)
C (18)	−1122 (7)	−418 (8)	3017 (5)	52 (2)
C (22)	−3729 (7)	4613 (9)	4433 (5)	54 (2)
C (43)	2312 (6)	7892 (7)	3136 (5)	45 (2)

**Table 3:** Bond lengths [Å] and angles [°] for **17a**.

Au (1)–Cl (1)	2.2876 (15)
Au (1)–C (1)	1.990 (6)
Si(4)–C (36)	1.871 (7)
Si(4)–C (42)	1.851 (8)
Si(4)–C (41)	1.874 (7)
Si(4)–C (43)	1.885 (7)
Si(3)–C (34)	1.878 (6)
Si(3)–C (38)	1.871 (8)
Si(3)–C (40)	1.877 (7)
Si(3)–C (39)	1.871 (7)
C (1)–N (1)	1.340 (8)
C (1)–N (2)	1.352 (8)
C (32)–C (37)	1.401 (9)
C (32)–C (33)	1.391 (9)
C (32)–C (27)	1.495 (9)
C (37)–H (37)	0.9500
C (37)–C (36)	1.407 (9)
C (36)–C (35)	1.406 (9)
C (34)–C (33)	1.394 (9)
C (34)–C (35)	1.409 (9)
C (33)–H (33)	0.9500
C (35)–H (35)	0.9500
C (38)-H (38A)	0.9800
C (38)-H (38B)	0.9800
C (38)-H (38C)	0.9800
C (40)-H (40A)	0.9800
C (40)-H (40B)	0.9800
C (40)-H (40C)	0.9800
C (42)-H (42A)	0.9800
C (42)-H (42B)	0.9800
C (42)-H (42C)	0.9800
C (39)-H (39A)	0.9800
C (39)-H (39B)	0.9800
C (39)-H (39C)	0.9800
C (41)-H (41A)	0.9800
C (41)-H (41B)	0.9800
C (41)-H (41C)	0.9800
N (1)–C (2)	1.387 (8)
N (1)–C (24)	1.480 (7)
C (2)–H (2)	0.9500
C (2)–C (3)	1.339 (9)
N (2)–C (3)	1.394 (7)
N (2)–C (4)	1.473 (7)
C (3)–H (3)	0.9500
C (26)–C (31)	1.384 (9)
C (26)–C (27)	1.404 (9)
C (26)–C (24)	1.523 (8)
C (31)–H (31)	0.9500
C (31)–C (30)	1.391 (10)
C (30)–H (30)	0.9500
C (30)–C (29)	1.368 (11)
C (27)–C (28)	1.395 (9)
C (24)–H (24)	1.0000
C (24)–C (25)	1.523 (8)
C (28)–H (28)	0.9500
C (28)–C (29)	1.388 (10)
C (29)–H (29)	0.9500
C (25)-H (25A)	0.9800
C (25)-H (25B)	0.9800
C (25)-H (25C)	0.9800
Si(2)–C (16)	1.877 (6)
Si(2)–C (23)	1.854 (7)
Si(2)–C (21)	1.866 (8)
Si(2)–C (22)	1.872 (8)
Si(1)–C (14)	1.894 (7)
Si(1)–C (19)	1.863 (8)
Si(1)–C (20)	1.871 (7)
Si(1)–C (18)	1.866 (9)
C (14)–C (15)	1.396 (9)
C (14)–C (13)	1.396 (9)
C (6)–C (11)	1.408 (8)
C (6)–C (7)	1.382 (8)
C (6)–C (4)	1.531 (8)
C (10)–H (10)	0.9500
C (10)–C (9)	1.386 (9)
C (10)–C (11)	1.394 (9)
C (17)–H (17)	0.9500
C (17)–C (16)	1.389 (8)
C (17)–C (12)	1.405 (8)
C (15)–H (15)	0.9500
C (15)–C (16)	1.408 (9)
C (13)–H (13)	0.9500
C (13)–C (12)	1.396 (9)
C (9)–H (9)	0.9500
C (9)–C (8)	1.387 (9)
C (5)-H (5A)	0.9800
C (5)-H (5B)	0.9800
C (5)-H (5C)	0.9800
C (5)–C (4)	1.522 (8)
C (11)–C (12)	1.487 (8)
C (7)–H (7)	0.9500
C (7)–C (8)	1.390 (9)
C (4)–H (4)	1.0000
C (8)–H (8)	0.9500
C (19)-H (19A)	0.9800
C (19)-H (19B)	0.9800
C (19)-H (19C)	0.9800
C (23)-H (23A)	0.9800
C (23)-H (23B)	0.9800
C (23)-H (23C)	0.9800
C (20)-H (20A)	0.9800
C (20)-H (20B)	0.9800
C (20)-H (20C)	0.9800
C (21)-H (21A)	0.9800
C (21)-H (21B)	0.9800
C (21)-H (21C)	0.9800
C (18)-H (18A)	0.9800
C (18)-H (18B)	0.9800
C (18)-H (18C)	0.9800
C (22)-H (22A)	0.9800
C (22)-H (22B)	0.9800
C (22)-H (22C)	0.9800
C (43)-H (43A)	0.9800
C (43)-H (43B)	0.9800
C (43)-H (43C)	0.9800
C (1)-Au (1)-Cl (1)	176.70 (17)
C (36)-Si(4)-C (41)	108.2 (3)
C (36)-Si(4)-C (43)	110.3 (3)
C (42)-Si(4)-C (36)	109.1 (3)
C (42)-Si(4)-C (41)	108.1 (4)
C (42)-Si(4)-C (43)	110.8 (4)
C (41)-Si(4)-C (43)	110.3 (4)
C (38)-Si(3)-C (34)	108.7 (3)
C (38)-Si(3)-C (40)	108.9 (4)
C (40)-Si(3)-C (34)	108.7 (3)
C (39)-Si(3)-C (34)	108.9 (3)
C (39)-Si(3)-C (38)	110.3 (4)
C (39)-Si(3)-C (40)	111.3 (4)
N (1)-C (1)-Au (1)	129.7 (4)
N (1)-C (1)-N (2)	105.8 (5)
N (2)-C (1)-Au (1)	124.4 (4)
C (37)-C (32)-C (27)	122.2 (6)
C (33)-C (32)-C (37)	119.0 (6)
C (33)-C (32)-C (27)	118.7 (6)
C (32)-C (37)-H (37)	119.5
C (32)-C (37)-C (36)	121.0 (6)
C (36)-C (37)-H (37)	119.5
C (37)-C (36)-Si(4)	123.4 (5)
C (35)-C (36)-Si(4)	119.2 (5)
C (35)-C (36)-C (37)	117.5 (6)
C (33)-C (34)-Si(3)	120.3 (5)
C (33)-C (34)-C (35)	116.4 (6)
C (35)-C (34)-Si(3)	123.3 (5)
C (32)-C (33)-C (34)	122.9 (6)
C (32)-C (33)-H (33)	118.6
C (34)-C (33)-H (33)	118.6
C (36)-C (35)-C (34)	123.2 (6)
C (36)-C (35)-H (35)	118.4
C (34)-C (35)-H (35)	118.4
Si(3)-C (38)-H (38A)	109.5
Si(3)-C (38)-H (38B)	109.5
Si(3)-C (38)-H (38C)	109.5
H (38A)-C (38)-H (38B)	109.5
H (38A)-C (38)-H (38C)	109.5
H (38B)–C (38)-H (38C)	109.5
Si(3)-C (40)-H (40A)	109.5
Si(3)-C (40)-H (40B)	109.5
Si(3)-C (40)-H (40C)	109.5
H (40A)-C (40)-H (40B)	109.5
H (40A)-C (40)-H (40C)	109.5
H (40B)–C (40)-H (40C)	109.5
Si(4)-C (42)-H (42A)	109.5
Si(4)-C (42)-H (42B)	109.5
Si(4)-C (42)-H (42C)	109.5
H (42A)-C (42)-H (42B)	109.5
H (42A)-C (42)-H (42C)	109.5
H (42B)–C (42)-H (42C)	109.5
Si(3)-C (39)-H (39A)	109.5
Si(3)-C (39)-H (39B)	109.5
Si(3)-C (39)-H (39C)	109.5
H (39A)-C (39)-H (39B)	109.5
H (39A)-C (39)-H (39C)	109.5
H (39B)–C (39)-H (39C)	109.5
Si(4)-C (41)-H (41A)	109.5
Si(4)-C (41)-H (41B)	109.5
Si(4)-C (41)-H (41C)	109.5
H (41A)-C (41)-H (41B)	109.5
H (41A)-C (41)-H (41C)	109.5
H (41B)–C (41)-H (41C)	109.5
C (1)-N (1)-C (2)	110.3 (5)
C (1)-N (1)-C (24)	124.6 (5)
C (2)-N (1)-C (24)	125.1 (5)
N (1)-C (2)-H (2)	126.3
C (3)-C (2)-N (1)	107.4 (5)
C (3)-C (2)-H (2)	126.3
C (1)-N (2)-C (3)	110.2 (5)
C (1)-N (2)-C (4)	123.3 (5)
C (3)-N (2)-C (4)	126.1 (5)
C (2)-C (3)-N (2)	106.4 (5)
C (2)-C (3)-H (3)	126.8
N (2)-C (3)-H (3)	126.8
C (31)-C (26)-C (27)	118.9 (6)
C (31)-C (26)-C (24)	121.4 (6)
C (27)-C (26)-C (24)	119.5 (5)
C (26)-C (31)-H (31)	119.6
C (26)-C (31)-C (30)	120.8 (7)
C (30)-C (31)-H (31)	119.6
C (31)-C (30)-H (30)	119.7
C (29)-C (30)-C (31)	120.6 (6)
C (29)-C (30)-H (30)	119.7
C (26)-C (27)-C (32)	121.6 (6)
C (28)-C (27)-C (32)	118.9 (6)
C (28)-C (27)-C (26)	119.4 (6)
N (1)-C (24)-C (26)	108.8 (5)
N (1)-C (24)-H (24)	107.8
N (1)-C (24)-C (25)	108.4 (5)
C (26)-C (24)-H (24)	107.8
C (25)-C (24)-C (26)	115.8 (5)
C (25)-C (24)-H (24)	107.8
C (27)-C (28)-H (28)	119.6
C (29)-C (28)-C (27)	120.8 (7)
C (29)-C (28)-H (28)	119.6
C (30)-C (29)-C (28)	119.4 (7)
C (30)-C (29)-H (29)	120.3
C (28)-C (29)-H (29)	120.3
C (24)-C (25)-H (25A)	109.5
C (24)-C (25)-H (25B)	109.5
C (24)-C (25)-H (25C)	109.5
H (25A)-C (25)-H (25B)	109.5
H (25A)-C (25)-H (25C)	109.5
H (25B)–C (25)-H (25C)	109.5
C (23)-Si(2)-C (16)	109.6 (3)
C (23)-Si(2)-C (21)	109.2 (4)
C (23)-Si(2)-C (22)	110.0 (4)
C (21)-Si(2)-C (16)	109.2 (4)
C (21)-Si(2)-C (22)	110.2 (4)
C (22)-Si(2)-C (16)	108.6 (3)
C (19)-Si(1)-C (14)	110.3 (3)
C (19)-Si(1)-C (20)	109.7 (4)
C (19)-Si(1)-C (18)	109.2 (4)
C (20)-Si(1)-C (14)	109.0 (3)
C (18)-Si(1)-C (14)	108.4 (3)
C (18)-Si(1)-C (20)	110.1 (4)
C (15)-C (14)-Si(1)	122.5 (5)
C (15)-C (14)-C (13)	117.9 (6)
C (13)-C (14)-Si(1)	119.5 (5)
C (11)-C (6)-C (4)	119.6 (5)
C (7)-C (6)-C (11)	119.1 (5)
C (7)-C (6)-C (4)	121.3 (5)
C (9)-C (10)-H (10)	119.3
C (9)-C (10)-C (11)	121.5 (6)
C (11)-C (10)-H (10)	119.3
C (16)-C (17)-H (17)	119.0
C (16)-C (17)-C (12)	122.0 (6)
C (12)-C (17)-H (17)	119.0
C (14)-C (15)-H (15)	118.6
C (14)-C (15)-C (16)	122.7 (6)
C (16)-C (15)-H (15)	118.6
C (14)-C (13)-H (13)	119.3
C (12)-C (13)-C (14)	121.5 (6)
C (12)-C (13)-H (13)	119.3
C (17)-C (16)-Si(2)	121.8 (5)
C (17)-C (16)-C (15)	117.2 (6)
C (15)-C (16)-Si(2)	120.9 (5)
C (10)-C (9)-H (9)	120.3
C (10)-C (9)-C (8)	119.3 (6)
C (8)-C (9)-H (9)	120.3
H (5A)-C (5)-H (5B)	109.5
H (5A)-C (5)-H (5C)	109.5
H (5B)–C (5)-H (5C)	109.5
C (4)-C (5)-H (5A)	109.5
C (4)-C (5)-H (5B)	109.5
C (4)-C (5)-H (5C)	109.5
C (6)-C (11)-C (12)	122.2 (5)
C (10)-C (11)-C (6)	118.9 (5)
C (10)-C (11)-C (12)	118.9 (5)
C (6)-C (7)-H (7)	119.2
C (6)-C (7)-C (8)	121.5 (6)
C (8)-C (7)-H (7)	119.2
N (2)-C (4)-C (6)	111.3 (5)
N (2)-C (4)-C (5)	110.8 (5)
N (2)-C (4)-H (4)	107.4
C (6)-C (4)-H (4)	107.4
C (5)-C (4)-C (6)	112.2 (5)
C (5)-C (4)-H (4)	107.4
C (17)-C (12)-C (11)	119.9 (5)
C (13)-C (12)-C (17)	118.7 (6)
C (13)-C (12)-C (11)	121.4 (5)
C (9)-C (8)-C (7)	119.6 (6)
C (9)-C (8)-H (8)	120.2
C (7)-C (8)-H (8)	120.2
Si(1)-C (19)-H (19A)	109.5
Si(1)-C (19)-H (19B)	109.5
Si(1)-C (19)-H (19C)	109.5
H (19A)-C (19)-H (19B)	109.5
H (19A)-C (19)-H (19C)	109.5
H (19B)–C (19)-H (19C)	109.5
Si(2)-C (23)-H (23A)	109.5
Si(2)-C (23)-H (23B)	109.5
Si(2)-C (23)-H (23C)	109.5
H (23A)-C (23)-H (23B)	109.5
H (23A)-C (23)-H (23C)	109.5
H (23B)–C (23)-H (23C)	109.5
Si(1)-C (20)-H (20A)	109.5
Si(1)-C (20)-H (20B)	109.5
Si(1)-C (20)-H (20C)	109.5
H (20A)-C (20)-H (20B)	109.5
H (20A)-C (20)-H (20C)	109.5
H (20B)–C (20)-H (20C)	109.5
Si(2)-C (21)-H (21A)	109.5
Si(2)-C (21)-H (21B)	109.5
Si(2)-C (21)-H (21C)	109.5
H (21A)-C (21)-H (21B)	109.5
H (21A)-C (21)-H (21C)	109.5
H (21B)–C (21)-H (21C)	109.5
Si(1)-C (18)-H (18A)	109.5
Si(1)-C (18)-H (18B)	109.5
Si(1)-C (18)-H (18C)	109.5
H (18A)-C (18)-H (18B)	109.5
H (18A)-C (18)-H (18C)	109.5
H (18B)–C (18)-H (18C)	109.5
Si(2)-C (22)-H (22A)	109.5
Si(2)-C (22)-H (22B)	109.5
Si(2)-C (22)-H (22C)	109.5
H (22A)-C (22)-H (22B)	109.5
H (22A)-C (22)-H (22C)	109.5
H (22B)–C (22)-H (22C)	109.5
Si(4)-C (43)-H (43A)	109.5
Si(4)-C (43)-H (43B)	109.5
Si(4)-C (43)-H (43C)	109.5
H (43A)-C (43)-H (43B)	109.5
H (43A)-C (43)-H (43C)	109.5
H (43B)–C (43)-H (43C)	109.5

**Table 4:** Crystal data and structure refinement for **18a**.

Identification code	s1	
Empirical formula	C45H64 Au Cl N2 Si4	
Formula weight	977.75	
Temperature	100 (2) K	
Wavelength	1.54178 Å	
Crystal system	Tetragonal	
Space group	*P*4_**3**_2_**1**_2	
Unit cell dimensions	a = 12.2766 (2) Å	α = 90°.
	b = 12.2766 (2) Å	β = 90°.
	c = 64.5450 (12) Å	γ = 90°.
Volume	9727.9 (4) Å^3^	
Z	8	
Density (calculated)	1.335 Mg/m^3^	
Absorption coefficient	7.356 mm^−1^	
F (000)	4000	
Crystal size	0.040 × 0.040 × 0.010 mm^3^	
Theta range for data collection	2.738–74.183°.	
Index ranges	−13 ≤ h<=14, −14 ≤ k<=15, −76 ≤ l<=79	
Reflections collected	139555	
Independent reflections	9852 [R (int) = 0.1584]	
Completeness to theta = 67.679°	100.0%	
Absorption correction	Semi-empirical from equivalents	
Max. and min. transmission	0.7538 and 0.6255	
Refinement method	Full-matrix least-squares on F^2^	
Data/restraints/parameters	9852/0/493	
Goodness-of-fit on F^2^	1.028	
Final R indices [I > 2sigma(I)]	R1 = 0.0373, w*R*2 = 0.0720	
R indices (all data)	R1 = 0.0523, w*R*2 = 0.0771	
Absolute structure parameter	−0.038 (6)	
Extinction coefficient	0.000299 (18)	
Largest diff. peak and hole	0.775 and −0.702 e.Å^−3^

**Table 5:** Atomic coordinates (x 10^4^) and equivalent isotropic displacement parameters (Å^2^x 10^3^).for **18a**. U (eq) is defined as one third of the trace of the orthogonalized U^ij^ tensor.

	x	y	z	U (eq)
Au (1)	3202 (1)	1505 (1)	4107 (1)	27 (1)
Cl (1)	4449 (2)	170 (2)	4172 (1)	33 (1)
Si(1)	3202 (2)	−328 (2)	4749 (1)	29 (1)
Si(2)	6059 (2)	3345 (2)	4622 (1)	34 (1)
Si(4)	6854 (2)	3261 (2)	2970 (1)	41 (1)
Si(3)	7074 (2)	1168 (2)	3748 (1)	40 (1)
N (007)	1303 (4)	3079 (5)	4138 (1)	27 (1)
N (008)	2128 (4)	3188 (5)	3846 (1)	26 (1)
C (18)	2558 (6)	1885 (6)	4702 (1)	28 (2)
C (30)	976 (6)	1793 (6)	3288 (1)	32 (2)
C (28)	2509 (6)	2399 (6)	3498 (1)	25 (2)
C (2)	1300 (6)	3952 (6)	3841 (1)	30 (2)
C (1)	2155 (6)	2658 (6)	4030 (1)	29 (2)
C (5)	−103 (6)	2007 (6)	4326 (1)	33 (2)
C (3)	779 (6)	3874 (6)	4023 (1)	32 (2)
C (33)	3225 (5)	1904 (5)	3355 (1)	26 (1)
C (29)	1391 (6)	2329 (6)	3460 (1)	29 (2)
C (17)	3452 (6)	1160 (5)	4706 (1)	27 (1)
C (14)	3745 (5)	3395 (6)	4638 (1)	28 (2)
C (13)	2697 (6)	2999 (6)	4662 (1)	27 (2)
C (16)	4489 (6)	1606 (6)	4676 (1)	29 (2)
C (25)	2949 (6)	3019 (6)	3682 (1)	26 (2)
C (34)	4434 (6)	1999 (6)	3367 (1)	28 (2)
C (39)	4983 (6)	2478 (6)	3198 (1)	29 (2)
C (31)	1675 (6)	1297 (6)	3151 (1)	34 (2)
C (38)	6111 (6)	2582 (6)	3187 (1)	31 (2)
C (36)	6216 (5)	1675 (6)	3527 (1)	31 (2)
C (26)	3440 (7)	4123 (6)	3622 (1)	33 (2)
C (11)	1656 (7)	4601 (6)	4788 (1)	33 (2)
C (7)	889 (6)	3590 (6)	4503 (1)	29 (2)
C (22)	2499 (7)	−502 (6)	5005 (1)	38 (2)
C (32)	2792 (6)	1351 (6)	3186 (1)	29 (2)
C (35)	5074 (6)	1606 (6)	3529 (1)	31 (2)
C (10)	773 (7)	5298 (7)	4788 (1)	36 (2)
C (15)	4668 (6)	2732 (6)	4646 (1)	30 (2)
C (9)	−56 (7)	5143 (6)	4646 (1)	37 (2)
C (37)	6703 (6)	2169 (6)	3356 (1)	33 (2)
C (8)	6 (6)	4292 (6)	4506 (1)	33 (2)
C (12)	1731 (6)	3734 (5)	4648 (1)	26 (1)
C (4)	950 (6)	2673 (6)	4344 (1)	28 (2)
C (21)	7097 (7)	2243 (7)	4597 (1)	42 (2)
C (27)	3942 (8)	4717 (7)	3806 (1)	50 (2)
C (41)	6806 (9)	−309 (7)	3789 (1)	54 (2)
C (23)	4514 (7)	−1094 (7)	4743 (1)	42 (2)
C (40)	6768 (8)	1974 (7)	3984 (1)	48 (2)
C (6)	30 (7)	999 (7)	4196 (1)	40 (2)
C (24)	2281 (7)	−853 (6)	4544 (1)	37 (2)
C (42)	8523 (7)	1396 (11)	3678 (1)	73 (3)
C (20)	6351 (8)	4160 (8)	4860 (1)	52 (2)
C (19)	6108 (8)	4262 (7)	4391 (1)	51 (2)
C (45)	7818 (9)	4264 (8)	3080 (2)	65 (3)
C (44)	7682 (11)	2241 (9)	2826 (2)	90 (5)
C (43)	5864 (10)	3986 (16)	2802 (2)	135 (8)

**Table 6:** Bond lengths [Å] and angles [°] for **18a**.

Au (1)–C (1)	1.976 (7)
Au (1)–Cl (1)	2.2813 (16)
Si(1)–C (24)	1.858 (8)
Si(1)–C (23)	1.865 (9)
Si(1)–C (17)	1.873 (7)
Si(1)–C (22)	1.874 (8)
Si(2)–C (19)	1.866 (9)
Si(2)–C (21)	1.866 (8)
Si(2)–C (20)	1.871 (8)
Si(2)–C (15)	1.873 (8)
Si(4)–C (45)	1.849 (10)
Si(4)–C (43)	1.854 (13)
Si(4)–C (44)	1.862 (11)
Si(4)–C (38)	1.872 (7)
Si(3)–C (42)	1.855 (10)
Si(3)–C (40)	1.858 (8)
Si(3)–C (41)	1.862 (10)
Si(3)–C (36)	1.876 (7)
N (007)–C (1)	1.359 (9)
N (007)–C (3)	1.383 (9)
N (007)–C (4)	1.487 (8)
N (008)–C (1)	1.357 (9)
N (008)–C (2)	1.383 (8)
N (008)–C (25)	1.475 (8)
C (18)–C (13)	1.401 (10)
C (18)–C (17)	1.414 (10)
C (18)–H (18)	0.9500
C (30)–C (31)	1.374 (10)
C (30)–C (29)	1.388 (10)
C (30)–H (30)	0.9500
C (28)–C (29)	1.397 (10)
C (28)–C (33)	1.413 (9)
C (28)–C (25)	1.509 (9)
C (2)–C (3)	1.340 (10)
C (2)–H (2)	0.9500
C (5)–C (6)	1.503 (10)
C (5)–C (4)	1.533 (10)
C (5)-H (5A)	0.9900
C (5)-H(5B)	0.9900
C (3)–H (3)	0.9500
C (33)–C (32)	1.389 (9)
C (33)–C (34)	1.491 (9)
C (29)–H (29)	0.9500
C (17)–C (16)	1.399 (10)
C (14)–C (13)	1.383 (10)
C (14)–C (15)	1.396 (10)
C (14)–H (14)	0.9500
C (13)–C (12)	1.494 (9)
C (16)–C (15)	1.412 (11)
C (16)–H (16)	0.9500
C (25)–C (26)	1.532 (10)
C (25)–H (25)	1.0000
C (34)–C (35)	1.396 (10)
C (34)–C (39)	1.408 (10)
C (39)–C (38)	1.392 (10)
C (39)–H (39)	0.9500
C (31)–C (32)	1.391 (10)
C (31)–H (31)	0.9500
C (38)–C (37)	1.404 (10)
C (36)–C (37)	1.396 (10)
C (36)–C (35)	1.405 (10)
C (26)–C (27)	1.522 (10)
C (26)-H (26A)	0.9900
C (26)-H (26B)	0.9900
C (11)–C (10)	1.381 (11)
C (11)–C (12)	1.399 (9)
C (11)–H (11)	0.9500
C (7)–C (8)	1.386 (10)
C (7)–C (12)	1.408 (10)
C (7)–C (4)	1.522 (10)
C (22)-H (22A)	0.9800
C (22)-H (22B)	0.9800
C (22)-H (22C)	0.9800
C (32)–H (32)	0.9500
C (35)–H (35)	0.9500
C (10)–C (9)	1.382 (11)
C (10)–H (10)	0.9500
C (9)–C (8)	1.385 (10)
C (9)–H (9)	0.9500
C (37)–H (37)	0.9500
C (8)–H (8)	0.9500
C (4)–H (4)	1.0000
C (21)-H (21A)	0.9800
C (21)-H (21B)	0.9800
C (21)-H (21C)	0.9800
C (27)-H (27A)	0.9800
C (27)-H (27B)	0.9800
C (27)-H (27C)	0.9800
C (41)-H (41A)	0.9800
C (41)-H (41B)	0.9800
C (41)-H (41C)	0.9800
C (23)-H (23A)	0.9800
C (23)-H (23B)	0.9800
C (23)-H (23C)	0.9800
C (40)-H (40A)	0.9800
C (40)-H (40B)	0.9800
C (40)-H (40C)	0.9800
C (6)-H (6A)	0.9800
C (6)-H (6B)	0.9800
C (6)-H (6C)	0.9800
C (24)-H (24A)	0.9800
C (24)-H (24B)	0.9800
C (24)-H (24C)	0.9800
C (42)-H (42A)	0.9800
C (42)-H (42B)	0.9800
C (42)-H (42C)	0.9800
C (20)-H (20A)	0.9800
C (20)-H (20B)	0.9800
C (20)-H (20C)	0.9800
C (19)-H (19A)	0.9800
C (19)-H (19B)	0.9800
C (19)-H (19C)	0.9800
C (45)-H (45A)	0.9800
C (45)-H (45B)	0.9800
C (45)-H (45C)	0.9800
C (44)-H (44A)	0.9800
C (44)-H (44B)	0.9800
C (44)-H (44C)	0.9800
C (43)-H (43A)	0.9800
C (43)-H (43B)	0.9800
C (43)-H (43C)	0.9800
C (1)-Au (1)-Cl (1)	175.9 (2)
C (24)-Si(1)-C (23)	109.6 (4)
C (24)-Si(1)-C (17)	109.4 (3)
C (23)-Si(1)-C (17)	110.3 (4)
C (24)-Si(1)-C (22)	107.9 (4)
C (23)-Si(1)-C (22)	111.1 (4)
C (17)-Si(1)-C (22)	108.5 (3)
C (19)-Si(2)-C (21)	110.3 (4)
C (19)-Si(2)-C (20)	109.1 (4)
C (21)-Si(2)-C (20)	109.1 (4)
C (19)-Si(2)-C (15)	109.8 (4)
C (21)-Si(2)-C (15)	109.8 (4)
C (20)-Si(2)-C (15)	108.7 (4)
C (45)-Si(4)-C (43)	108.9 (7)
C (45)-Si(4)-C (44)	106.9 (5)
C (43)-Si(4)-C (44)	112.9 (8)
C (45)-Si(4)-C (38)	108.6 (4)
C (43)-Si(4)-C (38)	109.4 (4)
C (44)-Si(4)-C (38)	110.0 (4)
C (42)-Si(3)-C (40)	108.2 (5)
C (42)-Si(3)-C (41)	110.5 (5)
C (40)-Si(3)-C (41)	111.4 (4)
C (42)-Si(3)-C (36)	107.8 (4)
C (40)-Si(3)-C (36)	109.4 (4)
C (41)-Si(3)-C (36)	109.4 (4)
C (1)-N (007)-C (3)	110.6 (5)
C (1)-N (007)-C (4)	123.7 (6)
C (3)-N (007)-C (4)	125.5 (6)
C (1)-N (008)-C (2)	111.1 (6)
C (1)-N (008)-C (25)	123.0 (6)
C (2)-N (008)-C (25)	125.7 (6)
C (13)-C (18)-C (17)	121.5 (7)
C (13)-C (18)-H (18)	119.2
C (17)-C (18)-H (18)	119.2
C (31)-C (30)-C (29)	119.7 (7)
C (31)-C (30)-H (30)	120.2
C (29)-C (30)-H (30)	120.2
C (29)-C (28)-C (33)	118.0 (6)
C (29)-C (28)-C (25)	121.4 (6)
C (33)-C (28)-C (25)	120.5 (6)
C (3)-C (2)-N (008)	106.6 (6)
C (3)-C (2)-H (2)	126.7
N (008)-C (2)-H (2)	126.7
N (008)-C (1)-N (007)	104.4 (6)
N (008)-C (1)-Au (1)	125.5 (5)
N (007)-C (1)-Au (1)	130.1 (5)
C (6)-C (5)-C (4)	112.9 (6)
C (6)-C (5)-H (5A)	109.0
C (4)-C (5)-H (5A)	109.0
C (6)-C (5)-H (5B)	109.0
C (4)-C (5)-H (5B)	109.0
H (5A)-C (5)-H (5B)	107.8
C (2)-C (3)-N (007)	107.3 (6)
C (2)-C (3)-H (3)	126.4
N (007)-C (3)-H (3)	126.4
C (32)-C (33)-C (28)	119.0 (6)
C (32)-C (33)-C (34)	117.3 (6)
C (28)-C (33)-C (34)	123.5 (6)
C (30)-C (29)-C (28)	122.1 (7)
C (30)-C (29)-H (29)	119.0
C (28)-C (29)-H (29)	119.0
C (16)-C (17)-C (18)	117.2 (6)
C (16)-C (17)-Si(1)	123.5 (5)
C (18)-C (17)-Si(1)	119.3 (6)
C(13)-C (14)-C (15)	123.0 (7)
C (13)-C (14)-H (14)	118.5
C (15)-C (14)-H (14)	118.5
C (14)-C (13)-C (18)	118.5 (7)
C (14)-C (13)-C (12)	121.2 (6)
C (18)-C (13)-C (12)	120.2 (6)
C (17)-C (16)-C (15)	122.9 (7)
C (17)-C (16)-H (16)	118.5
C (15)-C (16)-H (16)	118.5
N (008)-C (25)-C (28)	112.9 (6)
N (008)-C (25)-C (26)	109.0 (6)
C (28)-C (25)-C (26)	112.9 (5)
N (008)-C (25)-H (25)	107.3
C (28)-C (25)-H (25)	107.3
C (26)-C (25)-H (25)	107.3
C (35)-C (34)-C (39)	117.0 (6)
C (35)-C (34)-C (33)	124.8 (6)
C (39)-C (34)-C (33)	118.0 (6)
C (38)-C (39)-C (34)	123.5 (7)
C (38)-C (39)-H (39)	118.2
C (34)-C (39)-H (39)	118.2
C (30)-C (31)-C (32)	119.4 (7)
C (30)-C (31)-H (31)	120.3
C (32)-C (31)-H (31)	120.3
C (39)-C (38)-C (37)	116.3 (7)
C (39)-C (38)-Si(4)	124.2 (6)
C (37)-C (38)-Si(4)	119.4 (5)
C (37)-C (36)-C (35)	117.3 (7)
C (37)-C (36)-Si(3)	120.3 (5)
C (35)-C (36)-Si(3)	122.4 (5)
C (27)-C (26)-C (25)	112.7 (6)
C (27)-C (26)-H (26A)	109.0
C (25)-C (26)-H (26A)	109.0
C (27)-C (26)-H (26B)	109.0
C (25)-C (26)-H (26B)	109.0
H (26A)-C (26)-H (26B)	107.8
C (10)-C (11)-C (12)	121.5 (7)
C (10)-C (11)-H (11)	119.3
C (12)-C (11)-H (11)	119.3
C (8)-C (7)-C (12)	119.1 (6)
C (8)-C (7)-C (4)	120.5 (7)
C (12)-C (7)-C (4)	120.4 (6)
Si(1)-C (22)-H (22A)	109.5
Si(1)-C (22)-H (22B)	109.5
H (22A)-C (22)-H (22B)	109.5
Si(1)-C (22)-H (22C)	109.5
H (22A)-C (22)-H (22C)	109.5
H (22B)–C (22)-H (22C)	109.5
C (33)-C (32)-C (31)	121.8 (6)
C (33)-C (32)-H (32)	119.1
C (31)-C (32)-H (32)	119.1
C (34)-C (35)-C (36)	122.4 (6)
C (34)-C (35)-H (35)	118.8
C (36)-C (35)-H (35)	118.8
C (11)-C (10)-C (9)	119.6 (7)
C (11)-C (10)-H (10)	120.2
C (9)-C (10)-H (10)	120.2
C (14)-C (15)-C (16)	116.7 (7)
C (14)-C (15)-Si(2)	120.1 (6)
C (16)-C (15)-Si(2)	123.1 (6)
C (10)-C (9)-C (8)	119.8 (7)
C (10)-C (9)-H (9)	120.1
C (8)-C (9)-H (9)	120.1
C (36)-C (37)-C (38)	123.4 (7)
C (36)-C (37)-H (37)	118.3
C (38)-C (37)-H (37)	118.3
C (9)-C (8)-C (7)	121.4 (7)
C (9)-C (8)-H (8)	119.3
C (7)-C (8)-H (8)	119.3
C (11)-C (12)-C (7)	118.6 (6)
C (11)-C (12)-C (13)	118.3 (6)
C (7)-C (12)-C (13)	123.1 (6)
N (007)-C (4)-C (7)	111.6 (6)
N (007)-C (4)-C (5)	110.8 (5)
C (7)-C (4)-C (5)	113.9 (6)
N (007)-C (4)-H (4)	106.7
C (7)-C (4)-H (4)	106.7
C (5)-C (4)-H (4)	106.7
Si(2)-C (21)-H (21A)	109.5
Si(2)-C (21)-H (21B)	109.5
H (21A)-C (21)-H (21B)	109.5
Si(2)-C (21)-H (21C)	109.5
H (21A)-C (21)-H (21C)	109.5
H (21B)–C (21)-H (21C)	109.5
C (26)-C (27)-H (27A)	109.5
C (26)-C (27)-H (27B)	109.5
H (27A)-C (27)-H (27B)	109.5
C (26)-C (27)-H (27C)	109.5
H (27A)-C (27)-H (27C)	109.5
H (27B)–C (27)-H (27C)	109.5
Si(3)-C (41)-H (41A)	109.5
Si(3)-C (41)-H (41B)	109.5
H (41A)-C (41)-H (41B)	109.5
Si(3)-C (41)-H (41C)	109.5
H (41A)-C (41)-H (41C)	109.5
H (41B)–C (41)-H (41C)	109.5
Si(1)-C (23)-H (23A)	109.5
Si(1)-C (23)-H (23B)	109.5
H (23A)-C (23)-H (23B)	109.5
Si(1)-C (23)-H (23C)	109.5
H (23A)-C (23)-H (23C)	109.5
H (23B)–C (23)-H (23C)	109.5
Si(3)-C (40)-H (40A)	109.5
Si(3)-C (40)-H (40B)	109.5
H (40A)-C (40)-H (40B)	109.5
Si(3)-C (40)-H (40C)	109.5
H (40A)-C (40)-H (40C)	109.5
H (40B)–C (40)-H (40C)	109.5
C (5)-C (6)-H (6A)	109.5
C (5)-C (6)-H (6B)	109.5
H (6A)-C (6)-H (6B)	109.5
C (5)-C (6)-H (6C)	109.5
H (6A)-C (6)-H (6C)	109.5
H (6B)–C (6)-H (6C)	109.5
Si(1)-C (24)-H (24A)	109.5
Si(1)-C (24)-H (24B)	109.5
H (24A)-C (24)-H (24B)	109.5
Si(1)-C (24)-H (24C)	109.5
H (24A)-C (24)-H (24C)	109.5
H (24B)–C (24)-H (24C)	109.5
Si(3)-C (42)-H (42A)	109.5
Si(3)-C (42)-H (42B)	109.5
H (42A)-C (42)-H (42B)	109.5
Si(3)-C (42)-H (42C)	109.5
H (42A)-C (42)-H (42C)	109.5
H (42B)–C (42)-H (42C)	109.5
Si(2)-C (20)-H (20A)	109.5
Si(2)-C (20)-H (20B)	109.5
H (20A)-C (20)-H (20B)	109.5
Si(2)-C (20)-H (20C)	109.5
H (20A)-C (20)-H (20C)	109.5
H (20B)–C(20)-H (20C)	109.5
Si(2)-C (19)-H (19A)	109.5
Si(2)-C (19)-H (19B)	109.5
H (19A)-C (19)-H (19B)	109.5
Si(2)-C (19)-H (19C)	109.5
H (19A)-C (19)-H (19C)	109.5
H (19B)–C (19)-H (19C)	109.5
Si(4)-C (45)-H (45A)	109.5
Si(4)-C (45)-H (45B)	109.5
H (45A)-C (45)-H (45B)	109.5
Si(4)-C (45)-H (45C)	109.5
H (45A)-C (45)-H (45C)	109.5
H (45B)–C (45)-H (45C)	109.5
Si(4)-C (44)-H (44A)	109.5
Si(4)-C (44)-H (44B)	109.5
H (44A)-C (44)-H (44B)	109.5
Si(4)-C (44)-H (44C)	109.5
H (44A)-C (44)-H (44C)	109.5
H (44B)–C (44)-H (44C)	109.5
Si(4)-C (43)-H (43A)	109.5
Si(4)-C (43)-H (43B)	109.5
H (43A)-C (43)-H (43B)	109.5
Si(4)-C (43)-H (43C)	109.5
H (43A)-C (43)-H (43C)	109.5
H (43B)–C (43)-H (43C)	109.5

**Table 7:** Crystal data and structure refinement for **24**.

Identification code	s1	
Empirical formula	C26H35 N O5 S	
Formula weight	473.61	
Temperature	100.0 K	
Wavelength	0.71073 Å	
Crystal system	Orthorhombic	
Space group	*Pbca*	
Unit cell dimensions	a = 14.2401 (6)Å	α = 90°.
	b = 9.6319 (4) Å	β = 90°.
	c = 35.1349 (14) Å	γ = 90°.
Volume	4819.1 (3) Å^3^	
Z	8	
Density (calculated)	1.306 Mg/m^3^	
Absorption coefficient	0.172 mm^−1^	
F (000)	2032	
Crystal size	0.77 × 0.52 × 0.15 mm^3^	
Theta range for data collection	2.319–36.369°.	
Index ranges	−23 ≤ h<=23, −16 ≤ k<=16, −58 ≤ l<=58	
Reflections collected	229040	
Independent reflections	11692 [R (int) = 0.0830]	
Completeness to theta = 25.242°	99.9%	
Absorption correction	Semi-empirical from equivalents	
Max. and min. transmission	0.4383 and 0.3908	
Refinement method	Full-matrix least-squares on F^2^	
Data/restraints/parameters	11692/0/403	
Goodness-of-fit on F^2^	1.065	
Final R indices [I > 2sigma(I)]	R1 = 0.0421, w*R*2 = 0.0996	
R indices (all data)	R1 = 0.0634, w*R*2 = 0.1072	
Extinction coefficient	n/a	
Largest diff. peak and hole	0.650 and −0.514 e.Å^−3^

**Table 8:** Atomic coordinates (x 10^4^) and equivalent isotropic displacement parameters (Å^2^x 10^3^) for **24**. U (eq) is defined as one third of the trace of the orthogonalized U^ij^ tensor.

	x	y	z	U (eq)
S (1)	4600 (1)	4617 (1)	2155 (1)	11 (1)
O (1)	5322 (1)	2364 (1)	806 (1)	13 (1)
O (5)	4603 (1)	4299 (1)	2555 (1)	16 (1)
O (3)	3584 (1)	−313 (1)	1313 (1)	16 (1)
O (4)	3941 (1)	5649 (1)	2026 (1)	17 (1)
N (1)	4411 (1)	3125 (1)	1925 (1)	10 (1)
C (21)	6497 (1)	4202 (1)	2134 (1)	11 (1)
C (2)	4187 (1)	2012 (1)	1301 (1)	10 (1)
C (16)	5751 (1)	5043 (1)	2003 (1)	11 (1)
C (3)	4387 (1)	3340 (1)	1507 (1)	11 (1)
O (2)	4525 (1)	4306 (1)	648 (1)	22 (1)
C (4)	3544 (1)	2436 (1)	2066 (1)	13 (1)
C (6)	3426 (1)	1059 (1)	1462 (1)	11 (1)
C (5)	3468 (1)	996 (1)	1892 (1)	13 (1)
C (1)	4535 (1)	1677 (1)	961 (1)	11 (1)
C (11)	5249 (1)	3669 (1)	661 (1)	14 (1)
C (17)	5912 (1)	6172 (1)	1754 (1)	13 (1)
C (24)	6377 (1)	2981 (1)	2399 (1)	14 (1)
C (19)	7587 (1)	5590 (1)	1758 (1)	14 (1)
C (20)	7404 (1)	4508 (1)	2010 (1)	13 (1)
C (18)	6837 (1)	6404 (1)	1637 (1)	15 (1)
C (10)	4174 (1)	506 (1)	713 (1)	14 (1)
C (9)	3320 (1)	−158 (1)	921 (1)	17 (1)
C (7)	2504 (1)	1468 (1)	1270 (1)	15 (1)
C (25)	3875 (1)	1088 (1)	325 (1)	19 (1)
C (12)	6202 (1)	4158 (1)	514 (1)	18 (1)
C (23)	8564 (1)	5851 (1)	1611 (1)	19 (1)
C (8)	2456 (1)	762 (1)	946 (1)	19 (1)
C (22)	5176 (1)	7155 (1)	1602 (1)	22 (1)
C (26)	4937 (1)	−603 (1)	659 (1)	23 (1)
C (15)	6974 (1)	3834 (1)	804 (1)	26 (1)
C (13)	6395 (1)	3365 (2)	142 (1)	32 (1)
C (14)	6158 (1)	5716 (2)	438 (1)	38 (1)

**Table 9:** Bond lengths [Å] and angles [°] for **24**.

S (1)–O (5)	1.4391 (7)
S (1)–O (4)	1.4389 (7)
S (1)–N (1)	1.6694 (7)
S (1)–C (16)	1.7733 (9)
O (1)–C (1)	1.4121 (10)
O (1)–C (11)	1.3599 (11)
O (3)–C (6)	1.4383 (11)
O (3)–C (9)	1.4376 (11)
N (1)–C (3)	1.4834 (11)
N (1)–C (4)	1.4870 (11)
C (21)–C (16)	1.4133 (12)
C (21)–C (24)	1.5095 (12)
C (21)–C (20)	1.3953 (12)
C (2)–C (3)	1.4977 (12)
C (2)–C (6)	1.5281 (11)
C (2)–C (1)	1.3322 (11)
C (16)–C (17)	1.4134 (12)
O (2)–C (11)	1.2002 (11)
C (4)–C (5)	1.5192 (13)
C (6)–C (5)	1.5146 (12)
C (6)–C (7)	1.5275 (12)
C (1)–C (10)	1.5149 (12)
C (11)–C (12)	1.5281 (13)
C (17)–C (18)	1.3970 (13)
C (17)–C (22)	1.5103 (13)
C (19)–C (20)	1.3921 (13)
C (19)–C (18)	1.3914 (13)
C (19)–C (23)	1.5042 (13)
C (10)–C (9)	1.5561 (14)
C (10)–C (25)	1.5350 (13)
C (10)–C (26)	1.5350 (14)
C (9)–C (8)	1.5178 (15)
C (7)–C (8)	1.3295 (14)
C (12)–C (15)	1.5322 (15)
C (12)–C (13)	1.5364 (16)
C (12)–C (14)	1.5254 (17)
O (5)-S (1)-O (4)	117.08 (4)
O (5)-S (1)-N (1)	106.82 (4)
O (5)-S (1)-C (16)	109.94 (4)
O (4)-S (1)-N (1)	109.78 (4)
O (4)-S (1)-C (16)	110.38 (4)
N (1)-S (1)-C (16)	101.67 (4)
C (11)-O (1)-C (1)	121.07 (7)
C (9)-O (3)-C (6)	102.21 (7)
C (3)-N (1)-S (1)	111.19 (6)
C (3)-N (1)-C (4)	111.90 (6)
C (4)-N (1)-S (1)	110.90 (5)
C (16)-C (21)-C (24)	124.33 (8)
C (20)-C (21)-C (16)	118.14 (8)
C (20)-C (21)-C (24)	117.53 (8)
C (3)-C (2)-C (6)	117.99 (7)
C(1)-C (2)-C (3)	124.76 (8)
C (1)-C (2)-C (6)	116.66 (7)
C (21)-C (16)-S (1)	117.58 (6)
C (17)-C (16)-S (1)	120.98 (6)
C (17)-C (16)-C (21)	121.43 (8)
N (1)-C (3)-C (2)	111.36 (7)
N (1)-C (4)-C (5)	109.40 (7)
O (3)-C (6)-C (2)	107.93 (7)
O (3)-C (6)-C (5)	108.62 (7)
O (3)-C (6)-C (7)	102.22 (7)
C (5)-C (6)-C (2)	111.40 (7)
C (5)-C (6)-C (7)	118.91 (7)
C (7)-C (6)-C (2)	106.97 (7)
C (6)-C (5)-C (4)	111.65 (7)
O (1)-C (1)-C (10)	113.26 (7)
C (2)-C (1)-O (1)	121.93 (7)
C (2)-C (1)-C (10)	124.75 (8)
O (1)-C (11)-C (12)	110.05 (7)
O (2)-C (11)-O (1)	123.60 (8)
O (2)-C (11)-C (12)	126.33 (9)
C (16)-C (17)-C (22)	126.03 (8)
C (18)-C (17)-C (16)	117.21 (8)
C (18)-C (17)-C (22)	116.75 (8)
C (20)-C (19)-C (23)	121.07 (8)
C (18)-C (19)-C (20)	118.12 (8)
C (18)-C (19)-C (23)	120.80 (8)
C (19)-C (20)-C (21)	122.09 (8)
C (19)-C (18)-C (17)	122.98 (8)
C (1)-C (10)-C (9)	107.56 (7)
C (1)-C (10)-C (25)	109.47 (7)
C (1)-C (10)-C (26)	110.46 (8)
C (25)-C (10)-C (9)	110.43 (8)
C (26)-C (10)-C (9)	108.99 (8)
C (26)-C (10)-C (25)	109.90 (8)
O (3)-C (9)-C (10)	106.69 (7)
O (3)-C (9)-C (8)	102.53 (7)
C (8)-C (9)-C (10)	114.87 (8)
C (8)-C (7)-C (6)	106.81 (8)
C (11)-C (12)-C (15)	110.38 (8)
C (11)-C (12)-C (13)	107.12 (8)
C (15)-C (12)-C (13)	109.70 (10)
C (14)-C (12)-C (11)	108.99 (9)
C (14)-C (12)-C (15)	110.25 (10)
C (14)-C (12)-C (13)	110.35 (11)
C (7)-C (8)-C (9)	107.90 (8)

## 2 Experimental design, materials, and methods

All reactions were carried out under an inert nitrogen atmosphere with anhydrous solvents. Reagents were purchased and used without further purification unless otherwise stated. Reactions were monitored by thin-layer chromatography (TLC) carried out on Merck silica gel plates (60F-254; 0.25 mm) by using UV light as the visualizing agent and an acidic mixture of anisaldehyde, phosphomolybdic acid, ceric ammonium molybdate, or basic aqueous potassium permanganate (KMnO_4_) and heat as developing agents. Merck silica gel (60, particle size 0.043–0.063 mm) was used for flash column chromatography. NMR spectra were recorded on Bruker Av-500 and Av-300 instruments and calibrated by using residual undeuterated solvent as an internal reference (CHCl_3_: δ = 7.26 (^1^H), 77.16 ppm (^13^C)). Coupling constant in hertz (Hz). Single crystal X-ray diffraction (SCXRD) for **18a** and **19a** were collected on a Bruker SMART diffractometer equipped with an Apex II area detector using Mo-Kα radiation from a fine-focus sealed source tube with a focusing collimator (Bruker Nano, Inc., Madison, WI). SCXRD data for **24** was collected on a Bruker D8 Venture diffractometer equipped with a Photon 100 CMOS area detector using Mo-Kα radiation from a microfocus source (Bruker Nano). For all crystals, hemispheres of data were collected using strategies of scans about the omega and phi axes with frame widths of 0.5°. Data collection, unit cell determination, data reduction, absorption correction, and scaling were performed using the Bruker Apex3 software suite: Apex3, AXScale and SAINT, version 2017.3–0; Bruker AXS Inc.: Madison, WI, 2017. Enantiomeric excess was determined by HPLC (Lux® 5 μm Amylose-1, i-PrOH/hexane = 1/9, 0.5ml/min, 220nm).

### 2.1 Preparation of chiral sulfinamide intermediates

The chiral sulfinamide intermediates were synthesized according to previous report.[2].

**Figure:**
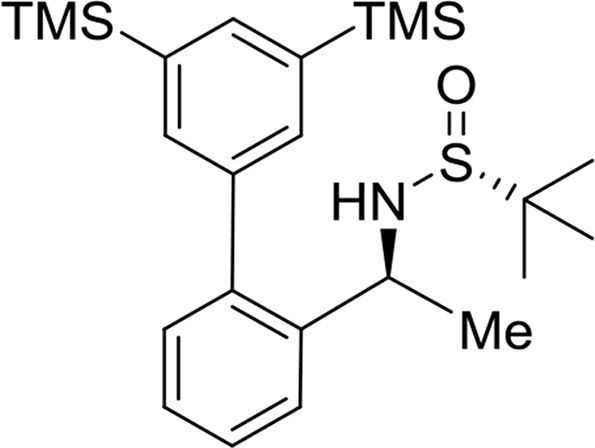

(*R*)-N-((*S*)-1-(3′,5′-bis(trimethylsilyl)-[1,1′-biphenyl]-2-yl)ethyl)-2-methylpropane-2-sulfinamide

Yield: 97%. ^1^H NMR (500MHz, CDCl_3_): δ 0.34 (s, 18H), 1.18 (s, 9H), 1.45 (d, *J* = 6.7, 3H), 3.29 (d, *J* = 3.9, 1H), 4.69–4.74 (m, 1H), 7.26 (dd, *J* = 7.7, 1.2, 1H), 7.33 (dt, *J* = 7.6, 1.2, 1H), 7.41 (dt, *J* = 7.6, 1.2, 1H), 7.48 (s, 2H), 7.54 (d, *J* = 7.6, 1H), 7.70 (s, 1H). ^13^C NMR (125MHz, CDCl_3_): δ −0.9, 22.6, 25.8, 51.3, 55.6, 126.6, 126.9, 127.8, 130.2, 134.6, 137.0, 139.3, 139.5, 141.7, 141.9. m/z (ESI-MS) calcd for [C_24_H_39_NOSSi_2_+H]: 446.2; found 446.2 [M+H]^+^.

**Figure:**
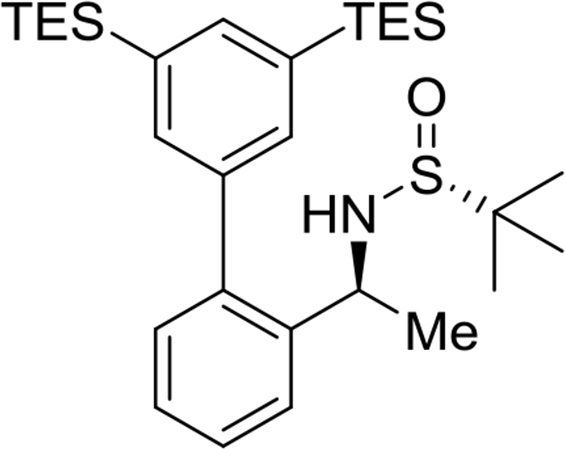

(*R*)-N-((*S*)-1-(3′,5′-bis(triethylsilyl)-[1,1′-biphenyl]-2-yl)ethyl)-2-methylpropane-2-sulfinamide

Yield: 99%. ^1^H NMR (500MHz, CDCl_3_): δ 0.83 (q, *J* = 7.9, 12H), 1.00 (t, *J* = 7.9, 18H), 1.13 (s, 9H), 1.46 (d, *J* = 6.8, 3H), 3.26 (d, *J* = 4.3, 1H), 4.70 (h, *J* = 6.5, 1H), 7.24 (d, *J* = 7.5, 1H), 7.31 (t, *J* = 7.4, 1H), 7.38–7.40 (m, 3H), 7.51 (d, *J* = 7.8, 1H), 7.63 (s, 1H). ^13^C NMR (125MHz, CDCl_3_): δ 3.5, 7.6, 22.6, 25.6, 51.6, 55.7, 126.3, 126.9, 127.7, 130.3, 135.2, 136.3, 139.1, 139.1, 141.9, 142.0. m/z (ESI-MS) calcd for [C_30_H_51_NOSSi_2_+H]: 530.3; found 530.2 [M+H]^+^.

**Figure:**
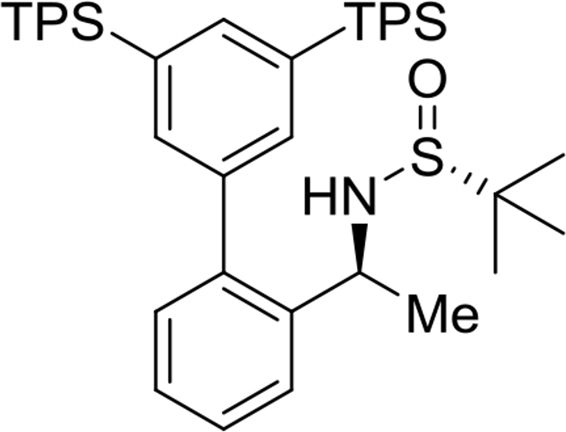

(*R*)-N-((*S*)-1-(3′,5′-bis(tripropylsilyl)-[1,1′-biphenyl]-2-yl)ethyl)-2-methylpropane-2-sulfinamide

Yield: 97%. ^1^H NMR (500MHz, CDCl_3_): δ 0.81–0.84 (m, 12H), 0.98 (t, *J* = 7.3, 18H), 1.13 (s, 9H), 1.36–1.44 (m, 12H), 1.47 (d, *J* = 6.7, 3H), 3.23 (d, *J* = 4.9, 1H), 4.66–4.71 (m, 1H), 7.24 (dd, *J* = 7.6, 1.4, 1H), 7.32 (dt, *J* = 7.5, 1.3, 1H), 7.37 (s, 2H), 7.40 (dt, *J* = 7.4, 1.4, 1H), 7.51 (dd, *J* = 7.9, 1.1, 1H), 7.61 (t, *J* = 1.1, 1H). ^13^C NMR (125MHz, CDCl_3_): δ 15.5, 17.6, 18.7, 22.6, 25.6, 51.8, 55.6, 126.3, 126.9, 127.7, 130.3, 135.1, 136.9, 138.9, 139.1, 142.0, 142.0.

**Figure:**
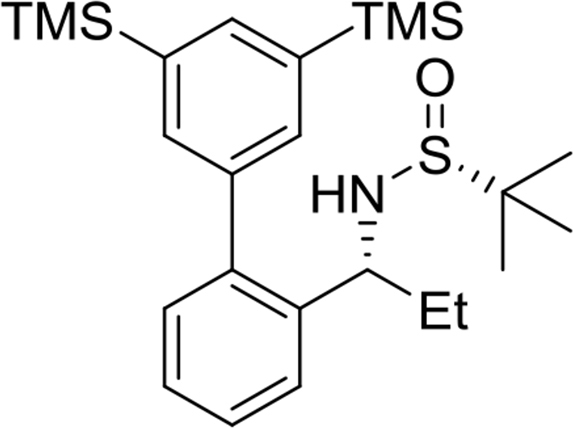

(*R*)-N-((*R*)-1-(3′,5′-bis(trimethylsilyl)-[1,1′-biphenyl]-2-yl)propyl)-2-methylpropane-2-sulfinamide

Yield: 93%. ^1^H NMR (500MHz, CDCl_3_): δ 0.36 (s, 18H), 0.75 (t, *J* = 7.4, 3H), 1.21 (s, 9H), 1.73–1.92 (m, 2H), 3.45 (d, *J* = 5.7, 1H), 4.38 (q, *J* = 7.0, 1H), 7.28 (dd, *J* = 7.6, 1.4, 1H), 7.33 (dt, *J* = 7.5, 1.4, 1H), 7.43 (dt, *J* = 7.2, 1.4, 1H), 7.49–7.51 (m, 3H), 7.72 (t, *J* = 1.1, 1H). ^13^C NMR (125MHz, CDCl_3_): δ −1.0, 10.4, 22.6, 30.9, 55.8, 57.1, 126.3, 127.0, 127.9, 130.2, 134.9, 136.8, 139.3, 139.5, 140.5, 142.4. m/z (ESI-MS) calcd for [C_25_H_41_NOSSi_2_+H]: 460.3; found 460.3 [M+H]^+^.

**Figure:**
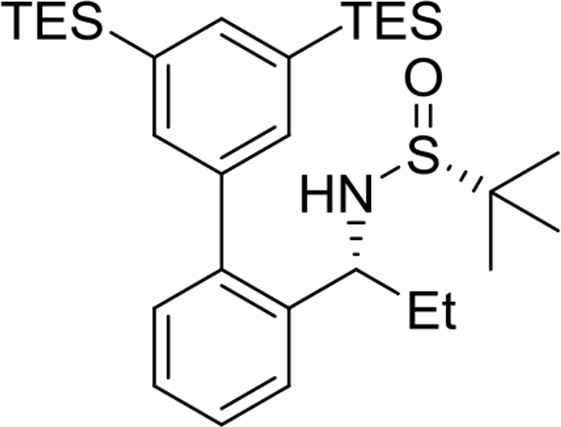

(*R*)-N-((*R*)-1-(3′,5′-bis(triethylsilyl)-[1,1′-biphenyl]-2-yl)propyl)-2-methylpropane-2-sulfinamide

Yield: 88%. ^1^H NMR (500MHz, CDCl_3_): δ 0.72 (t, *J* = 7.3, 3H), 0.83 (q, *J* = 7.6, 12H), 0.99 (t, *J* = 7.8, 18H), 1.16 (s, 9H), 1.68–1.81 (m, 2H), 3.43 (d, *J* = 6.7, 1H), 4.37 (q, *J* = 6.8, 1H), 7.24 (d, *J* = 7.7, 1H), 7.30 (t, *J* = 7.5, 1H), 7.39 (t, *J* = 7.8, 1H), 7.42 (s, 2H), 7.46 (d, *J* = 7.8, 1H), 7.62 (s, 1H). ^13^C NMR (125MHz, CDCl_3_): δ 3.5, 7.6, 10.7, 22.6, 31.1, 56.0, 57.7, 126.3, 127.0, 127.8, 130.3, 135.6, 136.0, 138.9, 139.4, 140.7, 142.4.

**Figure:**
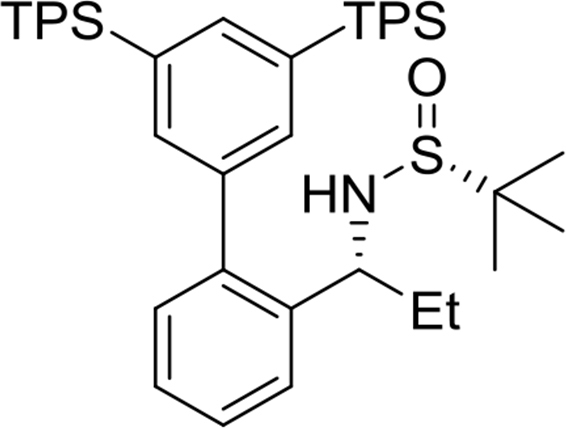

(*R*)-N-((*R*)-1-(3′,5′-bis(tripropylsilyl)-[1,1′-biphenyl]-2-yl)propyl)-2-methylpropane-2-sulfinamide

Yield: 90%. ^1^H NMR (500MHz, CDCl_3_): δ 0.73 (t, *J* = 7.4, 3H), 0.81–0.84 (m, 12H), 0.97 (t, *J* = 7.4, 18H), 1.16 (s, 9H), 1.35–1.43 (m, 12H), 1.66–1.82 (m, 2H), 3.41 (d, *J* = 7.0, 1H), 4.36 (q, *J* = 7.1, 1H), 7.23 (dd, *J* = 7.7, 1.3, 1H), 7.31 (dt, *J* = 7.5, 1.2, 1H), 7.38–7.41 (m, 3H), 7.46 (d, *J* = 7.8, 1H), 7.61 (s, 1H). ^13^C NMR (125MHz, CDCl_3_): δ 10.7, 15.4, 17.6, 18.6, 22.6, 31.2, 55.9, 57.8, 126.3, 127.0, 127.7, 130.3, 135.4, 136.7, 138.8, 139.3, 140.8, 142.4.

**Figure:**
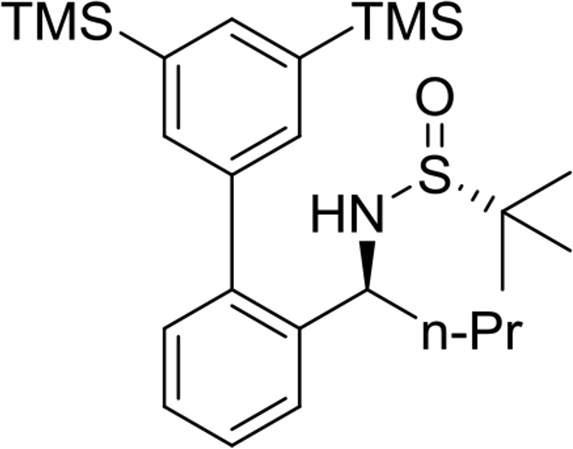

(*R*)-N-((*S*)-1-(3′,5′-bis(trimethylsilyl)-[1,1′-biphenyl]-2-yl)butyl)-2-methylpropane-2-sulfinamide

Yield: 50%. ^1^H NMR (500MHz, CDCl_3_): δ 0.37 (s, 18H), 0.76 (t, *J* = 7.4, 3H), 1.09–1.18 (m, 2H), 1.21 (s, 9H), 1.71–1.81 (m, 2H), 3.36 (d, *J* = 4.3, 1H), 4.56 (q, *J* = 4.2, 1H), 7.29 (d, *J* = 7.8, 1H), 7.35 (t, *J* = 7.5, 1H), 7.43 (t, *J* = 7.5, 1H), 7.49–7.51 (3H), 7.73 (s, 1H). ^13^C NMR (125MHz, CDCl_3_): δ −1.0, 13.8, 19.4, 22.6, 41.8, 55.4, 55.6, 126.70, 126.8, 127.7, 130.0, 134.8, 136.9, 139.3, 139.4, 140.8, 142.6.

**Figure:**
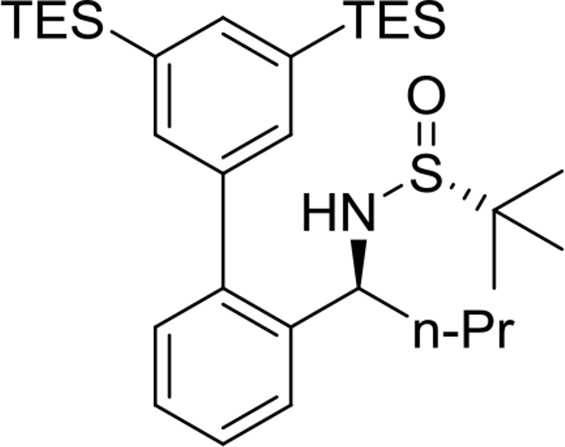

(*R*)-N-((*S*)-1-(3′,5′-bis(triethylsilyl)-[1,1′-biphenyl]-2-yl)butyl)-2-methylpropane-2-sulfinamide

Yield: 72%. ^1^H NMR (500MHz, CDCl_3_): δ 0.68 (t, *J* = 7.2, 3H), 0.84 (q, *J* = 7.9, 12H), 1.00 (t, *J* = 7.8, 18H), 1.05–1.13 (m, 2H), 1.16 (s, 9H), 1.63–1.76 (m, 2H), 3.30 (d, *J* = 5.0, 1H), 4.59 (q, *J* = 5.1, 1H), 7.23 (dd, *J* = 7.7, 1.07Hz), 7.30 (dt, *J* = 7.4, 1.2, 1H), 7.38 (dt, *J* = 7.7, 1.4, 1H), 7.40 (s, 2H), 7.46 (d, *J* = 7.7, 1H), 7.63 (s, 1H). ^13^C NMR (125MHz, CDCl_3_): δ 3.5, 7.6, 13.6, 19.4, 22.6, 41.7, 55.6, 55.7, 126.5, 126.7, 127.6, 130.2, 135.5, 136.2, 139.0, 139.3, 141.0, 142.5.

**Figure:**
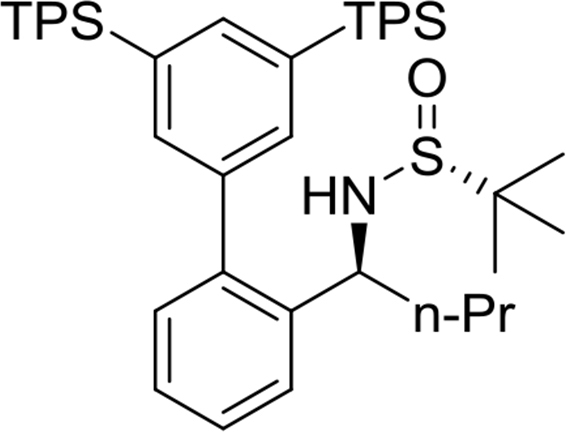

(*R*)-N-((*S*)-1-(3′,5′-bis(tripropylsilyl)-[1,1′-biphenyl]-2-yl)butyl)-2-methylpropane-2-sulfinamide

Yield: 86%. ^1^H NMR (500MHz, CDCl_3_): δ 0.71 (t, *J* = 7.3, 3H), 0.82–0.86 (m, 12H), 0.98 (t, *J* = 7.3, 18H), 1.08–1.23 (m, 2H), 1.17 (s, 9H), 1.37–1.44 (m, 12H), 1.63–1.76 (m, 2H), 3.29 (d, *J* = 5.2, 1H), 4.58 (q, *J* = 5.3, 1H), 7.23 (dd, *J* = 7.6, 1.1), 7.30 (dt, *J* = 7.5, 1.2, 1H), 7.37–7.40 (m, 3H), 7.46 (d, *J* = 7.9, 1H), 7.62 (s, 1H). ^13^C NMR (125MHz, CDCl_3_): δ 13.7, 15.4, 17.6, 18.6, 19.4, 22.6, 41.8, 55.6, 55.8, 126.5, 126.7, 127.6, 130.2, 135.3, 136.8, 138.9, 139.2, 141.1, 142.6.

### 2.2 Preparation of furan propargyl esters

**Figure:**
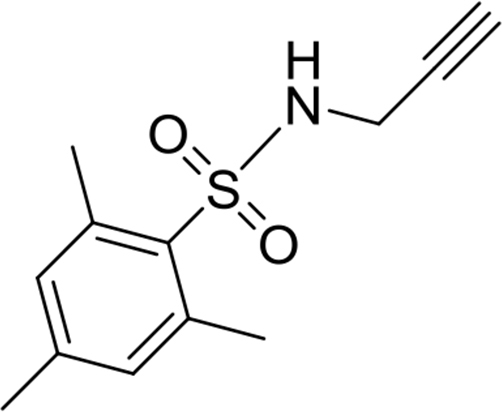

2,4,6-trimethyl-N-(prop-2-yn-1-yl)benzenesulfonamide

This compound was synthesized according to the procedure reported by Campolo et al.[3] Light brown solid. Yield: 93%. ^1^H NMR (500MHz, CDCl_3_): δ 2.09 (t, *J* = 2.6, 1H), 2.30 (s, 3H), 2.65 (s, 6H), 3.78–3.79 (m, 2H), 4.65 (s, 1H), 6.96 (s, 2H).

**Figure:**
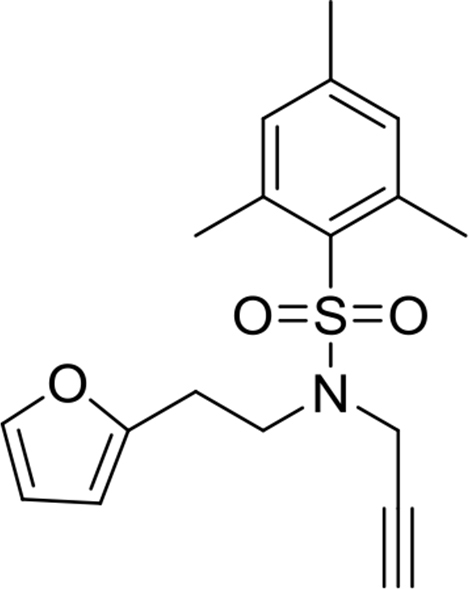

N-(2-(furan-2-yl)ethyl)-2,4,6-trimethyl-N-(prop-2-yn-1-yl)benzenesulfonamide

This compound was synthesized according to the procedure reported by Loshe et al.[4] Clear oil. Yield: 86%. ^1^H NMR (500MHz, CDCl_3_): δ 2.27 (t, *J* = 2.5, 1H), 2.30 (s, 3H), 2.88 (t, *J* = 7.2, 2H), 3.54 (t, *J* = 7.5, 2H), 4.02 (d, *J* = 2.5, 2H), 5.96 (dd, *J* = 3.2, 0.6, 1H), 6.23 (dd, *J* = 3.2, 1.9, 1H), 6.93 (s, 2H), 7.22 (dd, *J* = 2.1, 0.7, 1H). ^13^C NMR (125MHz, CDCl_3_): δ 21.1, 22.9, 26.5, 35.1, 44.4, 73.5, 77.7, 106.5, 110.4, 132.1, 132.3, 140.7, 141.6, 142.9, 152.2.

**Figure:**
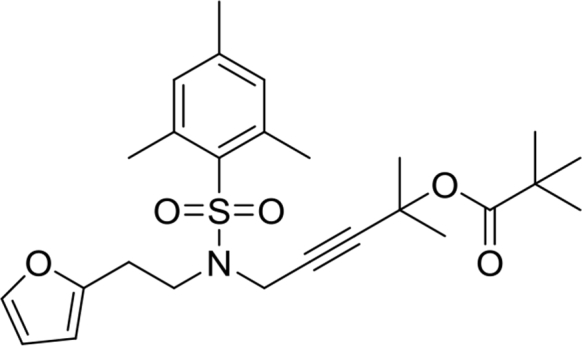

5-(N-(2-(furan-2-yl)ethyl)-2,4,6-trimethylphenylsulfonamido)-2-methylpent-3-yn-2-yl pivalate **23**

This compound was synthesized according to the procedure reported by Li et al.[5] Light yellow oil. Yield: 53%. ^1^H NMR (500MHz, CDCl_3_): δ 1.15 (s, 9H), 1.58 (s, 6H), 2.29 (s, 3H), 2.56 (s, 6H), 2.90 (t, *J* = 7.8, 2H), 3.53 (t, *J* = 7.6, 2H), 4.03 (s, 2H), 6.00 (dd, *J* = 3.2, 0.5, 1H), 6.23 (dd, *J* = 3.2, 1.8, 1H), 6.92 (s, 2H), 7.22 (dd, *J* = 1.8, 0.7, 1H). ^13^C NMR (125MHz, CDCl_3_): δ 21.1, 22.9, 26.6, 27.2, 28.8, 35.5, 39.2, 44.4, 71.4, 77.6, 86.6, 106.4, 110.3, 132.1, 132.7, 140.6, 141.5, 142.6, 152.5, 176.8.

**Figure:**
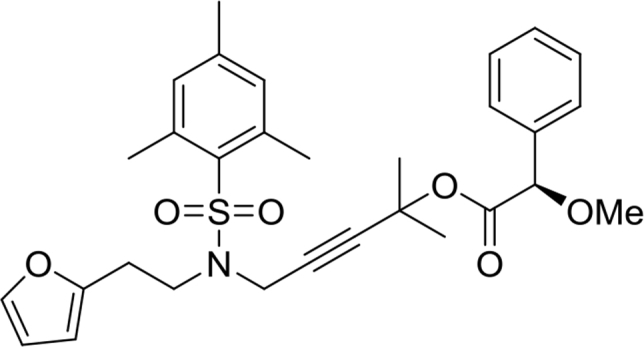

(*R*)-5-(N-(2-(furan-2-yl)ethyl)-2,4,6-trimethylphenylsulfonamido)-2-methylpent-3-yn-2-yl 2-methoxy-2-phenylacetate **28**

This compound was synthesized according to the procedure reported by Bao et al.[6] Light yellow oil. Yield: 86%. ^1^H NMR (500MHz, CDCl_3_): δ 1.54 (s, 3H), 1.60 (s, 3H), 2.30 (s, 3H), 2.54 (s, 6H), 2.84 (t, *J* = 7.2, 2H), 3.41 (s, 3H), 3.46 (dt, *J* = 7.3, 2.5, 2H), 3.99 (s, 2H), 4.69 (s, 1H), 5.99 (d, *J* = 3.1, 1H), 6.24 (q, *J* = 1.9, 1H), 6.92 (s, 2H), 7.22 (d, *J* = 1.2, 1H), 7.31–7.35 (m, 3H), 7.43 (dd, *J* = 8.0, 1.8, 2H). ^13^C NMR (125MHz, CDCl_3_): δ 20.9, 22.7, 26.3, 28.4, 28.7, 35.1, 44.3, 57.3, 73.0, 78.4, 82.7, 85.6, 106.4, 110.2, 127.2, 128.5, 128.6, 132.0, 132.5, 136.2, 140.4, 141.3, 142.5, 152.3, 168.8.

### 2.3 NMR spectra of the gold complex 17a-19c and cycloaddition product 24 and 29 are deposited in Mendeley Data (https://data.mendeley.com/datasets/zh4gp5x682/4). The methods used to acquire the spectra are illustrtated in Specifications Table

.

### 2.4 X-ray crystallography data of gold complex 17a, 18a and cycloaddition product 24. The methods used to acquire the data are illustrtated in Specifications Table

.

### 2.5 HPLC spectra of [4 + 3] cycloaddition reactions. The methods used to acquire the data are illustrtated in Specifications Table

.

### 2.6 Cartesian coordinates of optimized structure of 25 and 26. The methods used to acquire the data are illustrated in our original paper[1]

#### Conformation A of **25**

**Figure:**
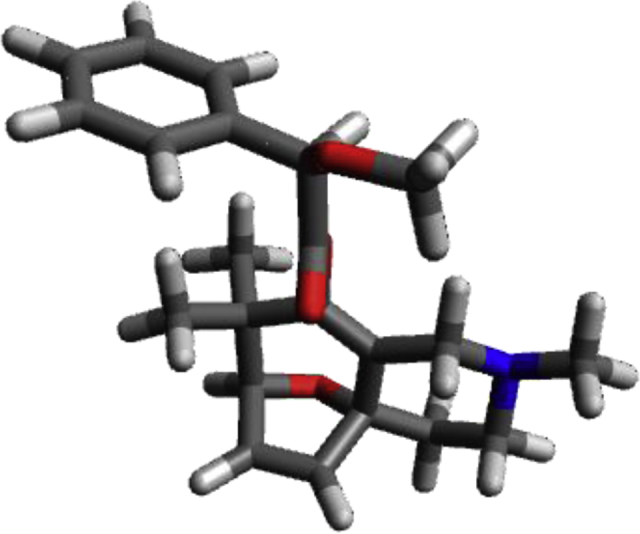

**Table:** 

C	−0.72548	−0.55653	0.29432
C	−1.92338	0.02460	0.17726
C	−3.10646	−0.91131	−0.11852
C	−3.01736	−1.28974	−1.60225
C	−2.23489	−2.36508	−1.68884
C	−1.80156	−2.73097	−0.27679
C	−0.46134	−2.05378	0.17204
H	−1.89406	−2.86387	−2.58877
H	−3.44694	−0.70861	−2.41083
O	0.42774	0.19865	0.60587
C	−2.16899	1.51122	0.17875
C	−4.55731	1.14847	0.14694
C	−4.42881	−0.35504	0.39775
H	−5.26167	−0.90062	−0.06010
H	−4.46167	−0.53355	1.47795
O	−2.84982	−2.17101	0.52224
H	−1.75333	−3.80821	−0.08422
C	1.01211	0.96718	−0.35514
O	0.59659	1.11278	−1.47755
C	2.29531	1.60911	0.21447
H	2.06864	1.93545	1.24201
O	2.71931	2.69023	−0.57885
C	3.40080	0.56664	0.24887
C	1.86974	3.82541	−0.52458
H	2.35516	4.60255	−1.11945
H	1.75484	4.18705	0.50950
H	0.88012	3.61559	−0.94856
C	3.71735	−0.10152	1.43572
C	4.71711	−1.07567	1.45083
C	5.40841	−1.38604	0.27859
C	5.09494	−0.71977	−0.90856
C	4.09255	0.24983	−0.92674
H	4.95939	−1.58556	2.37942
H	3.18335	0.14297	2.35062
H	6.18981	−2.14121	0.29035
H	5.63155	−0.95624	−1.82357
H	3.84664	0.77541	−1.84372
C	−0.06004	−2.62206	1.55095
H	0.81654	−2.09841	1.94559
H	−0.88375	−2.51449	2.26241
H	0.19174	−3.68669	1.46499
C	0.65631	−2.33200	−0.85489
H	0.45329	−1.84777	−1.81472
H	1.62299	−1.97235	−0.48977
H	0.74963	−3.41234	−1.02276
N	−3.45495	1.86283	0.78590
H	−2.10741	1.88288	−0.86741
H	−1.37455	2.01992	0.73569
H	−5.49769	1.50943	0.57871
H	−4.60638	1.35957	−0.94319
C	−3.65519	3.30344	0.77103
H	−2.84191	3.79460	1.31709
H	−4.59769	3.54996	1.27154
H	−3.68555	3.73128	−0.25121

#### Conformation B of **25**

**Figure:**
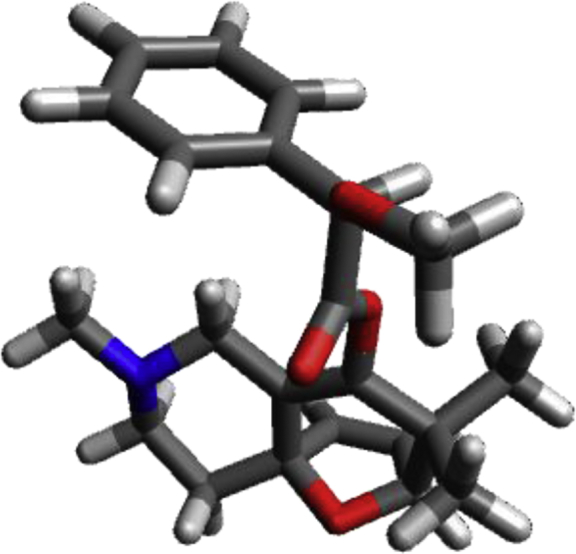

**Table:** 

C	1.10111	−0.76108	0.21770
C	1.44066	0.51326	0.00204
C	2.94022	0.80462	−0.15782
C	3.53721	0.80268	1.25638
C	3.87284	−0.45626	1.53874
C	3.53225	−1.30507	0.32209
C	2.08818	−1.91854	0.34529
H	4.26168	−0.84391	2.47352
H	3.57762	1.66829	1.90859
O	−0.24251	−1.10162	0.50563
C	0.50195	1.69173	0.06637
C	2.23714	3.16662	−0.73965
C	3.19965	2.01656	−1.04478
H	4.24122	2.33885	−0.93342
H	3.05200	1.70097	−2.08312
O	3.56636	−0.34747	−0.74108
H	4.26257	−2.09239	0.10684
C	1.95873	−2.87929	−0.85687
C	1.84646	−2.68901	1.66021
H	1.87895	−2.02619	2.53115
H	0.86952	−3.18110	1.64877
H	2.61141	−3.46479	1.78937
N	0.85406	2.73035	−0.90310
H	0.52074	2.10108	1.10275
H	−0.52841	1.37883	−0.11935
H	2.42383	3.99038	−1.43798
H	2.42260	3.56804	0.28091
C	−0.08282	3.84102	−0.83377
C	−1.11795	−1.30831	−0.51996
O	−0.83901	−1.29010	−1.69122
H	2.65556	−3.71951	−0.74195
H	2.18469	−2.35679	−1.78937
H	0.94746	−3.28970	−0.93397
C	−2.53255	−1.52952	0.05644
O	−3.37149	−2.16628	−0.87586
C	−3.01982	−3.51194	−1.15690
H	−3.79346	−3.89995	−1.82348
H	−3.00238	−4.11937	−0.23821
H	−2.04681	−3.58337	−1.65850
C	−4.18906	2.34773	0.99959
C	−4.16451	1.89256	−0.32145
C	−3.68345	1.54097	2.02019
C	−3.63409	0.63797	−0.62234
C	−3.15658	0.28330	1.72136
C	−3.13117	−0.17578	0.40112
H	−4.60392	3.32492	1.23213
H	−4.56114	2.51472	−1.11945
H	−3.70368	1.88652	3.05034
H	−2.76480	−0.34471	2.51760
H	−3.61437	0.27496	−1.64476
H	0.15618	4.57352	−1.61239
H	−0.07035	4.36222	0.14496
H	−1.10060	3.47523	−1.00615
H	−2.42782	−2.13098	0.97325

#### Conformation C of **26**

**Figure:**
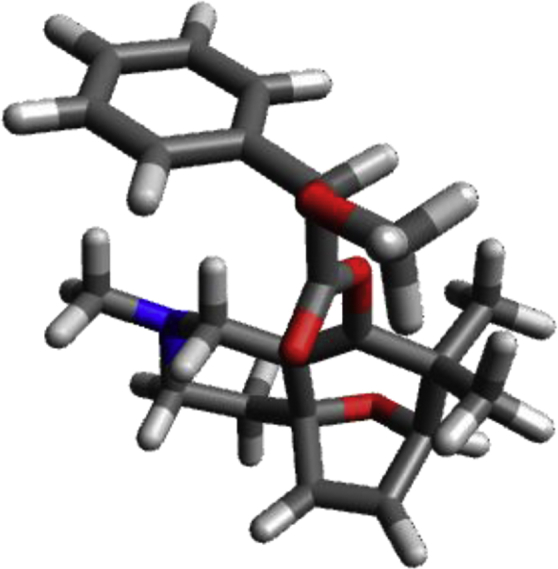

**Table:** 

C	1.10638	−0.68206	0.43566
C	1.41853	0.54935	0.02046
C	2.91759	0.86004	−0.11502
C	3.53767	−1.23168	0.41563
C	2.11447	−1.76807	0.79564
C	0.42811	1.58397	−0.44758
C	2.18481	3.23125	−0.62891
C	3.22101	2.34357	0.06395
N	0.84993	2.94599	−0.11154
H	2.41615	4.28336	−0.42693
H	2.23490	3.10102	−1.73180
H	0.28777	1.46942	−1.54493
H	−0.54922	1.40408	0.01059
H	3.20010	2.55557	1.13839
H	4.23065	2.55995	−0.30333
C	1.80575	−3.07406	0.03484
H	0.87075	−3.51911	0.39079
H	1.71265	−2.90262	−1.04156
H	2.60346	−3.80777	0.20523
C	2.07747	−2.02493	2.31812
H	2.39808	−1.13197	2.86190
H	1.06543	−2.28705	2.64394
H	2.74503	−2.85488	2.58249
C	3.39820	0.17811	−1.40211
C	3.75617	−1.06494	−1.08095
H	3.34982	0.62604	−2.38850
H	4.07782	−1.85427	−1.75062
O	3.62239	0.11547	0.89213
H	4.30439	−1.82973	0.92021
O	−0.23816	−1.07486	0.62591
C	−1.03293	−1.30076	−0.45775
O	−0.67192	−1.25876	−1.60714
C	−0.13495	3.91567	−0.56682
H	0.16482	4.92098	−0.25119
H	−1.10817	3.69011	−0.11753
H	−0.26309	3.92395	−1.66789
C	−2.47466	−1.58359	0.01550
O	−3.21131	−2.26317	−0.96995
C	−2.77896	−3.59327	−1.20854
H	−3.47498	−4.01850	−1.93523
H	−2.81350	−4.19451	−0.28640
H	−1.76409	−3.62414	−1.62414
C	−3.14924	0.27813	1.60185
C	−3.73612	1.51886	1.85457
C	−4.33284	2.23520	0.81510
C	−4.33868	1.70551	−0.47765
C	−3.74712	0.46846	−0.73361
C	−3.15294	−0.25400	0.30823
H	−3.73116	1.92199	2.86364
H	−4.79531	3.19868	1.01240
H	−2.68362	−0.27726	2.41194
H	−4.80422	2.25740	−1.28999
H	−3.74924	0.04897	−1.73395
H	−2.40893	−2.17581	0.94188

#### Conformation D of **26**

**Figure:**
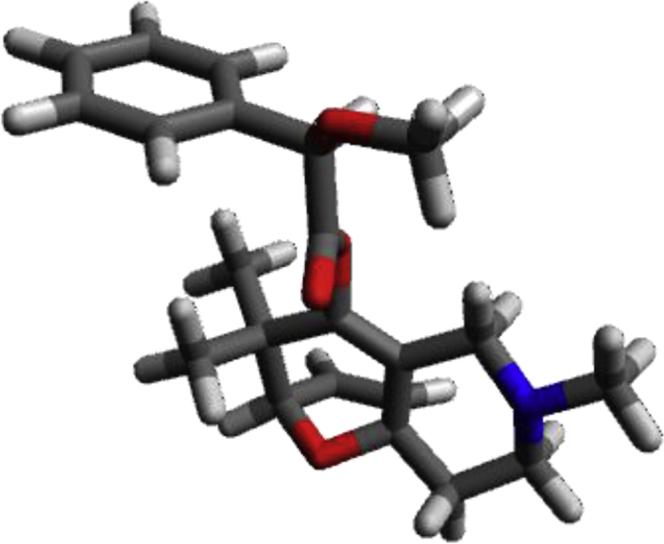

**Table:** 

C	0.73006	−0.53577	−0.13190
C	1.94317	0.02288	−0.17668
C	3.12770	−0.88382	0.19193
C	1.83724	−2.70789	0.38950
C	0.45070	−1.99168	0.23261
O	−0.40675	0.18081	−0.57439
C	2.24982	1.40216	−0.70393
C	4.55062	1.20144	0.00791
C	4.29593	−0.09772	0.77471
N	3.35412	2.03828	0.01558
H	5.36152	1.75642	0.49295
H	4.89129	0.98210	−1.02762
H	2.48472	1.32417	−1.79064
H	1.36985	2.04630	−0.62335
H	4.04156	0.15017	1.81070
H	5.19358	−0.72625	0.78702
C	−1.05335	1.01467	0.29070
O	−0.69277	1.27279	1.41030
C	−2.33013	1.55960	−0.38330
H	−2.09041	1.75268	−1.44088
O	−2.78626	2.72740	0.25594
C	−3.42030	0.50509	−0.29354
C	−1.93664	3.85249	0.09601
H	−2.44700	4.69405	0.57014
H	−1.77947	4.08487	−0.96932
H	−0.96517	3.70311	0.58337
C	−3.78362	−0.25136	−1.41158
C	−4.77372	−1.23076	−1.30846
C	−5.40787	−1.45727	−0.08604
C	−5.04783	−0.70230	1.03310
C	−4.05575	0.27254	0.93306
H	−5.05295	−1.81004	−2.18453
H	−3.29492	−0.07127	−2.36598
H	−6.18117	−2.21660	−0.00582
H	−5.54005	−0.87365	1.98672
H	−3.77332	0.86663	1.79637
C	−0.38990	−2.67282	−0.86806
H	−1.38809	−2.23039	−0.92669
H	0.08040	−2.58036	−1.85276
H	−0.50785	−3.74029	−0.64324
C	−0.29108	−2.07598	1.58512
H	0.31429	−1.63717	2.38196
H	−1.24950	−1.54928	1.54935
H	−0.49685	−3.12523	1.83281
C	3.39649	−1.77420	−1.02919
C	2.61708	−2.84905	−0.91005
H	4.04047	−1.50342	−1.85891
H	2.49350	−3.65876	−1.62019
O	2.67790	−1.84219	1.16009
H	1.70639	−3.64655	0.93822
C	3.61356	3.37074	−0.50513
H	2.71056	3.98462	−0.41423
H	3.92170	3.37312	−1.57042
H	4.40785	3.84670	0.07962
